# Discovery and comparative profiling of microRNAs in a sweet orange red-flesh mutant and its wild type

**DOI:** 10.1186/1471-2164-11-246

**Published:** 2010-04-17

**Authors:** Qiang Xu, Yuanlong Liu, Andan Zhu, Xiaomeng Wu, Junli Ye, Keqin Yu, Wenwu Guo, Xiuxin Deng

**Affiliations:** 1 Key Laboratory of Horticultural Plant Biology of Ministry of Education, Huazhong Agricultural University, Wuhan 430070, China; 2 National Key Laboratory of Crop Genetic Improvement, Huazhong Agricultural University, Wuhan 430070, China

## Abstract

**Background:**

Red-flesh fruit is absent from common sweet orange varieties, but is more preferred by consumers due to its visual attraction and nutritional properties. Our previous researches on a spontaneous red-flesh mutant revealed that the trait is caused by lycopene accumulation and is regulated by both transcriptional and post-transcriptional mechanisms. However, the knowledge on post-transcriptional regulation of lycopene accumulation in fruits is rather limited so far.

**Results:**

We used Illumina sequencing method to identify and quantitatively profile small RNAs on the red-flesh sweet orange mutant and its wild type. We identified 85 known miRNAs belonging to 48 families from sweet orange. Comparative profiling revealed that 51 known miRNAs exhibited significant expression differences between mutant (MT) and wild type (WT). We also identified 12 novel miRNAs by the presence of mature miRNAs and corresponding miRNA*s in the sRNA libraries. Comparative analysis showed that 9 novel miRNAs are differentially expressed between WT and MT. Target predictions of the 60 differential miRNAs resulted 418 target genes in sweet orange. GO and KEGG annotation revealed that high ranked miRNA-target genes are those implicated in transcription regulation, protein modification and photosynthesis. The expression profiles of target genes involved in carotenogenesis and photosynthesis were further confirmed to be complementary to the profiles of corresponding miRNAs in WT and MT.

**Conclusion:**

This study comparatively characterized the miRNAomes between the red-flesh mutant and the wild type, the results lay a foundation for unraveling the miRNA-mediated molecular processes that regulate lycopene accumulation in the sweet orange red-flesh mutant.

## Background

Sweet orange (*Citrus sinensis *[L.] Osbeck), one of the most important fruit crops worldwide, is responsible for 75% of citrus production used both as fresh fruit and processed juice [1]. Orange fruit is believed to contain many phytochemicals which are beneficial to human health [2]. Carotenoids are a group of the health-promoting products which may serve as antioxidants, vitamin A precursors, and cancer-preventing effectors [3]. Orange fruit is reported with more than 115 species of carotenoids [4], but lycopene are absent from common varieties [5-7]. Interest in lycopene metabolism and regulation is growing rapidly because a variety of epidemiological trials have suggested that higher intake of lycopene-containing fruits or foods are associated with decreased cardiovascular disease and prostate cancer risks [8]. So far, three sweet orange mutants with lycopene accumulation in the fruits were reported: Shara [9], Cara Cara [10] and the recently reported 'Hong Anliu' [11]. 'Hong Anliu' is a spontaneous bud mutant from its wild type 'Anliu' sweet orange. We found that lycopene in the 'Hong Anliu' mutant was 1000-fold higher than that in its wild type fruits, and in juice sacs the lycopene accumulation was coincided with increased expression of upstream carotenogenic genes and reduced expression of downstream genes [11]. Thereafter, transcriptional study including cDNA microarray in combination with suppression subtraction hybridization (SSH) was used to investigate gene expression changes in the mutant, and a total of 267 differentially expressed genes were detected [12]. More recently, massively parallel signature sequencing was applied to decipher the transcriptome changes between WT and MT; and the results indicated that partial impairment of lycopene downstream flux are critical for the formation of lycopene accumulation trait in the mutant [13]. Meanwhile, comparative proteomic analyses were performed between the mutant and its wild type. And the proteomic data, when considered in combination with transcriptional data, suggest that post-transcriptional regulations are involved in shaping the red-flesh trait of the orange mutant [14]. However, it remains largely unknown what kind of post-transcriptional mechanism was involved in the red-flesh mutant. So far, one interesting case has been reported in Arabidopsis where miRNA was proposed to regulate the expression of APETALA2 transcription factor (designated as RAP2.2) in controlling a key carotenoids biosynthesis gene (phytoene synthase gene) [15]. But to date, very little is known about miRNAs or miRNA-mediated molecular processes that regulate lycopene accumulation in plant fruits.

Small RNAs (sRNAs) are emerging as important regulators of biological processes on post-transcriptional level in most eukaryotes [16,17]. In plants, sRNAs are involved in a variety of activities that are essential for genome stability, development, and adaptive responses to biotic and abiotic stresses [16-21]. There is still increasing data for the implication of sRNAs in the regulation of other biological processes [22-24].

Based on difference of biogenesis and action, sRNAs have been mainly grouped into two categories, microRNA (miRNA) and short interfering RNA (siRNA) [21]. miRNAs distinguish from siRNAs by their biogenesis. miRNAs are cleaved from stem-loop precursor molecules that derive from single-stranded RNAs, while siRNAs are generated from double-strand RNA precursors [25]. In plants, miRNA regulates gene expression mostly on post-transcription level [17]. The majority of the miRNAs initially isolated from model plant *Arabidopsis thaliana *was found to be evolutionarily conserved across plant species [26]. Later, many researches suggested that the miRNA repertoire of plant species comprises a set of non-conserved miRNAs besides the conserved ones [27-29]. To date, 1894 miRNA sequences have been identified from 24 plant species, as revealed from miRNA registry database (miRBase v13.0; http://microrna.sanger.ac.uk). However, no citrus miRNAs have been found in this database so far.

Plant miRNAs have been identified mostly by two strategies: traditional Sanger sequencing of the small RNA libraries as for Arabidopsis [19,26], rice [30,31], and poplar [20]; and computational prediction of conserved miRNAs by searching for homologous sequences using EST or genomic sequences [18,32-36]. However, the computation-based approach is mostly limited to the discovery of conserved miRNAs; and the small-scale traditional sequencing approach identified mainly conserved miRNAs, as suggested by Morin et al. [37] and Moxon et al. [23]. Recently developed high-throughput sequencing technology has allowed the discovery of several non-conserved or lowly expressed miRNAs as well as quantification of the expression of miRNAs. This strategy has been successfully applied to both model plants [28,38-44], and non-model plants [23,37,45-50].

The criteria for miRNAs characterization and annotation in plants have been advanced since plants have relatively large and complex sRNA populations within which miRNAs are often a minority [51]. The minimum criteria include biogenesis criterion that miRNA precursors can be folded into stem-loop structure, and expression criterion that mature miRNAs should be detected by northern blotting, qRT-PCR or sequencing [52]. Proof of biogenesis requires data of the cloning or detection of miRNA* sequences, which is complementary to miRNA sequences in the precursor molecule [29,51]. Expression detection can also be done by recently developed qRT-PCR-based methods [53,54], where each miRNA is reverse-transcribed from total RNA using a specific stem-loop primer, followed by PCR amplification and detection. This method is believed to be a powerful alternative for sensitive, rapid, and cost-effective detection of miRNA [24,55]. Target prediction can be an ancillary criterion for miRNA annotation. Sequence complementarity between miRNAs and their target genes is very high in plants, which has made the search for plant miRNA target genes a straightforward process [18]. A number of prediction algorithms are available publicly for plant miRNA target prediction, such as miRU [56], WMD3 [57], and UEA plant sRNA toolkit [58].

Here we described the deep sequencing of sRNAs from a red-flesh sweet orange mutant and its wild type. Comparative profiling of the sRNA transcriptome between wild type and mutant revealed a set of 51 known and 9 novel miRNAs showing significant expression changes. The annotation of the potential targets of differential miRNAs indicated that high ranked genes are that implicated in biological processes including carotenoid biosynthesis, transcription regulation, and photosynthesis.

## Results

### Sequencing and annotation of sweet orange small RNAs

**Figure 1:**
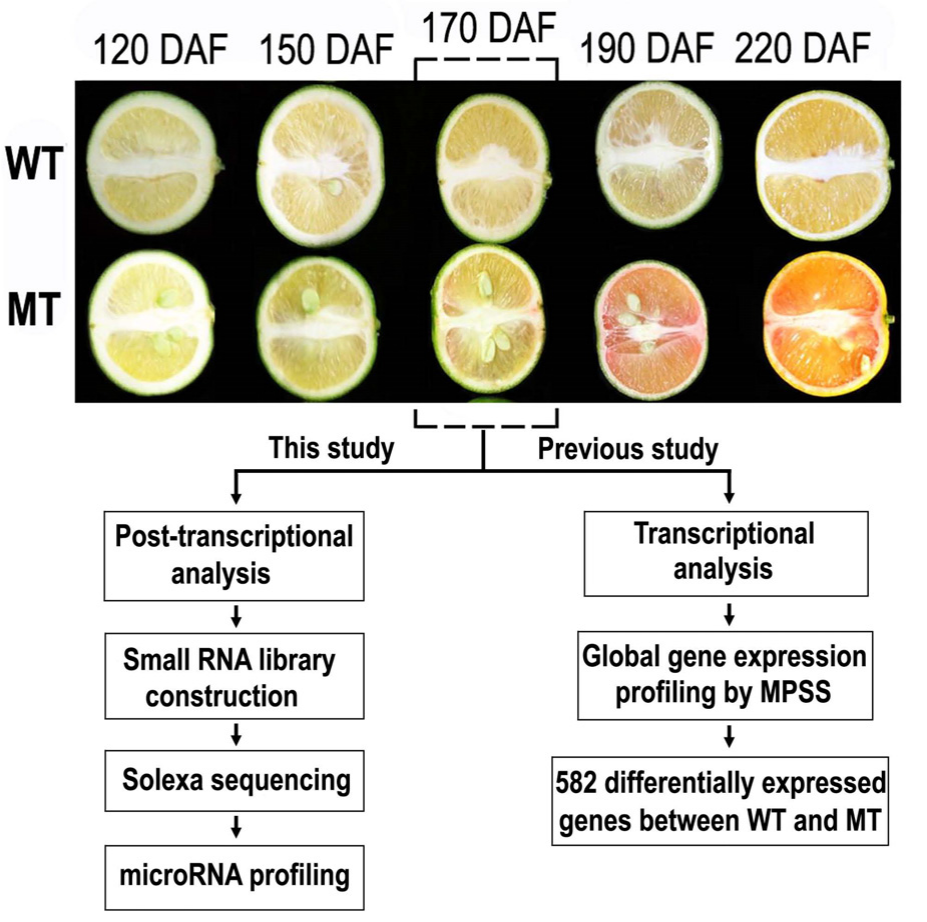
**Fruit development of the red-flesh mutant 'Hong anliu' sweet orange (MT) and its wild type (WT)**. The schematic presentation shows the strategy of transcriptional analysis by the Massive Parallel Signature Sequencing (previously) and post-transcriptional analysis by sRNA sequencing (this study) on the MT and WT. DAF stands for Days After Flowering.

**Figure 2:**
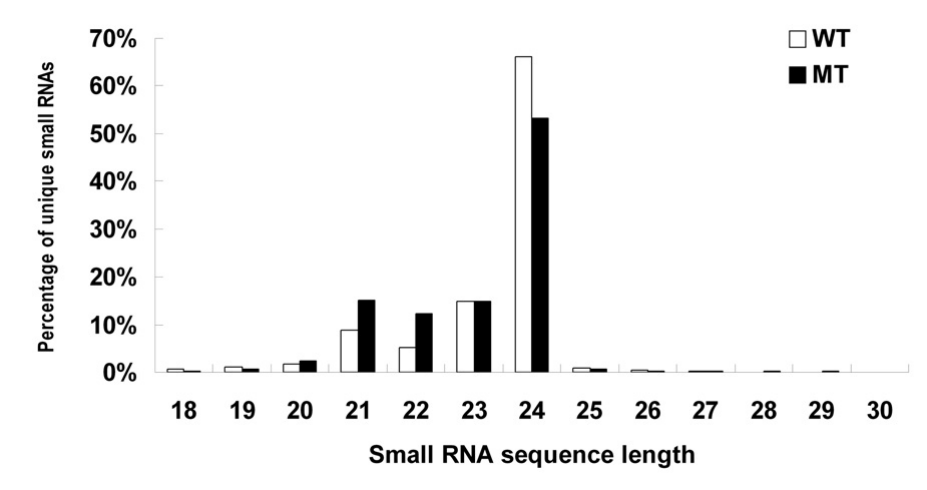
**Length distribution of sweet orange small RNA**.

Our previous studies showed that the stage of 170 days after flowering (DAF) is the critical stage for the phenotype of color break and global gene expression changes during fruit development in the mutant (Figure 1) [11,13]. In this study, sRNA libraries were generated from fruit pulps of 'Anliu' sweet orange (wild type, WT) and its red-flesh mutant (MT) 'Hong Anliu' at 170 DAF stage. Deep sequencing of small RNA libraries yielded 4,987,484 and 7,163,041 unfiltered sequence reads for WT and MT, respectively. The raw sRNA data were submitted to Gene Expression Omnibus (GEO) under accession no. GSE18207. After discarding low quality sequences containing ambiguous nucleotides, sequences shorter than 18 nucleotides, and sequences counts below 3 in both libraries, 1,790,395 (WT) and 3,345,746 (MT) reliable sequences were remained for analysis. These sequences represented 202,965 and 320,443 unique sRNA sequences in WT and MT, respectively. The distribution of unique sRNA length is summarized in Figure 2. The overall distribution pattern was similar between WT and MT, i.e. the majority of sRNAs (approximately 95%) were 21-24 nt in length with 24-nt sRNA as the major peak.

The unique sRNAs were further classified into different RNA categories (Table 1). Analysis showed that 10.8% (WT) and 9.9% (MT) of the unique sRNAs matched the *Citrus sinensis *unigenes (dataset from TIGR, the institute for genomic research). Detailed information was shown in Additional file 1 and 2. Further analysis using the sRNA sequences searching against Rfam database revealed that 1.7%, and 1.5% of the sequences for WT and MT, respectively, matched noncoding RNAs including rRNA, tRNA, snRNA and snoRNAs (Additional file 3 and 4). Searching the remaining sRNAs from WT and MT against miRBase v13.0 identified 0.4% and 0.3% of the unique sequences matched known miRNAs, which account 3.7% and 3.1% of the total sRNAs, respectively (Table 1).

**Table 1:** Summary of sRNAs sequences from WT and MT sweet orange

Category	Distinct signatures		Total signatures		Mean frequencies	
	**WT**	**MT**	**WT**	**MT**	**WT**	**MT**
matching protein-coding gene						
sense	13811 (6.80%)	18142 (5.66%)	244819 (13.67%)	346569 (10.36%)	17.73	19.10
antisense	8281 (4.08)	10651 (3.32%)	120749 (6.74%)	162839 (4.87%)	14.58	15.29
non-protein-coding RNAs						
snoRNA	13 (0.01%)	14 (0.01%)	471 (0.03%)	149 (0.01%)	36.23	10.64
snRNA	39 (0.02%)	104 (0.03%)	261 (0.01%)	859 (0.03%)	6.69	8.26
tRNA	647 (0.32%)	1301 (0.41%)	21932 (1.22%)	73778 (2.21%)	33.90	56.71
rRNA	2747 (1.35%)	3772 (1.18%)	59535 (3.33%)	99252 (2.97%)	21.67	26.31
miRNAs						
known	667 (0.33%)	802 (0.25%)	61993 (3.46%)	87455 (2.61%)	92.94	109.05
novel	83 (0.04%)	111 (0.03%)	4084 (0.23%)	11463 (0.34%)	49.20	103.27
other sRNAs	176677 (87.05%)	285546 (89.11%)	1276551 (71.30%)	2563382 (76.62%)	7.23	8.98
total	202965 (100%)	320443 (100%)	1790395 (100%)	3345746 (100%)	8.82	10.44

### Known miRNAs

We identified 85 miRNAs, belonging to 48 familes, in sweet orange by BLAST against the miRBase v13.0 using our combined (WT and MT) sRNA sequences (Additional file 5). The potential conserved miRNAs were used for BLASTN against sweet orange unigene dataset to search precursor sequences (Detail in Methods). Since the orange genome is largely unknown, we used orange unigene dataset (from TIGR, the institute for genomic research) and 1.2× coverage sequence of sweet orange (from NCBI) to predict RNA secondary structures. The sequences of perfect match with sRNA sequences were used for fold-back structure prediction by Mfold program [59]. The miRNA-containing fold-back structures were shown in Additional file 6.

To compare the miRNA abundance in different libraries, the count of each miRNA was normalized to transcripts per million (TPM). In the WT sRNA library, miR168, miR172 and miR156 were the most overrepresented families, with the abundance at 8,927, 4,754 and 3,566 TPM, respectively. These three miRNA families also frequently represented in MT sRNA library, but the difference is that the miR172 abundance ranked third (3,330 TPM) after miR156 (4,397 TPM) and miR168 (8,437 TPM) in the library. It is interesting that in both libraries miR168 was the most frequent miRNA which constituted nearly 32% of the miRNA transcriptome of sweet orange.

**Figure 3:**
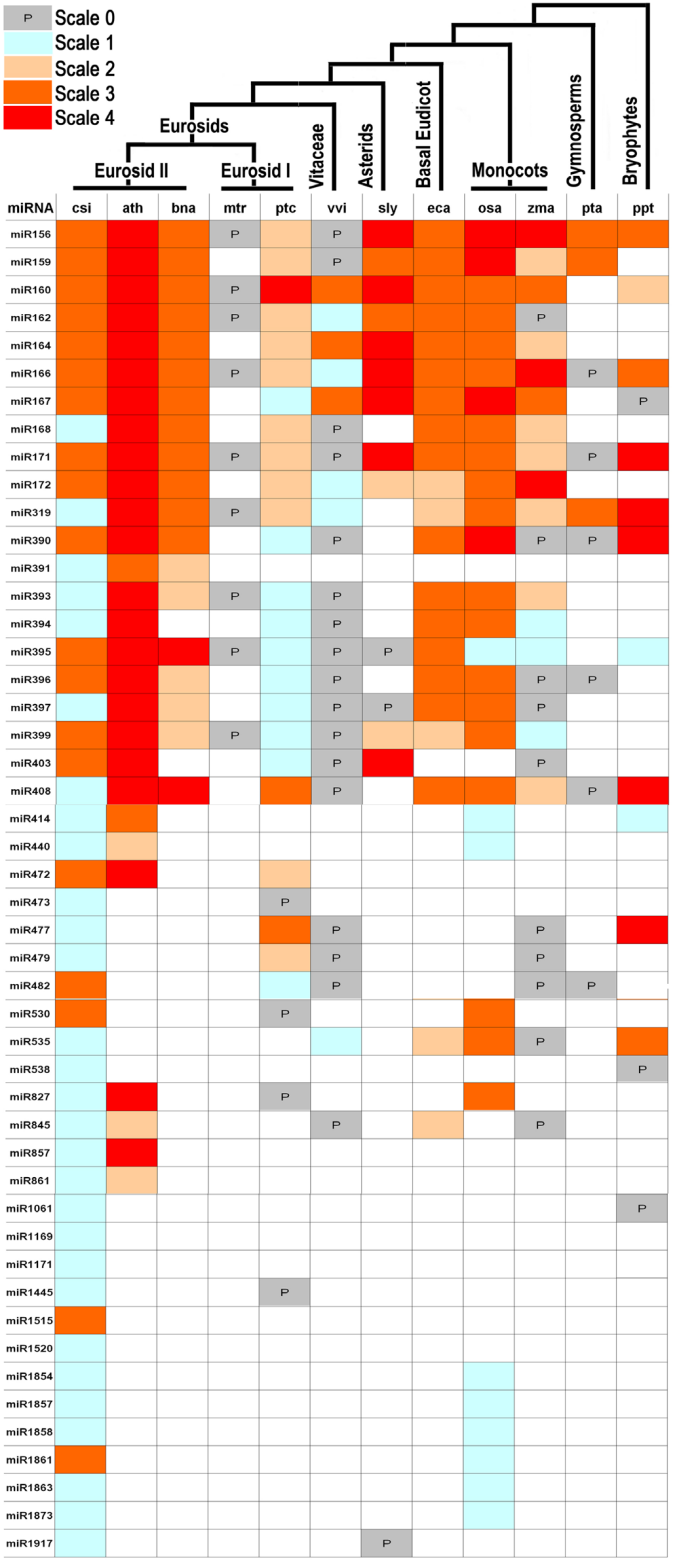
**Known miRNAs from sweet orange (*Citrus sinensis*), designated as csi on the first column, and their homologs in other plant species**. The picture is modified from Axtell et al. [45]. Scale 0, miRNA only predicted; scale 1, miRNA sequenced; scale 2, miRNA/miRNA* accumulation detected; scale 3, miRNA detected by small RNA blot or qRT-PCR; scale 4, miRNAs with validated targets. The general information is based on miRBase 13.0 statistics. Abbreviations: ath, *Arabidopsis thaliana*; bna, *Brassica napus*; csi, *Citrus sinensis*; eca, *Eschscholzia californica*; mtr, *Medicago truncatula*; osa, *Oryza sativa*; pta, *Pinus taeda*; ptc, *Populus trichocarpa*; ppt, *Physcomitrella patens*; sly; *Solanum lycopersicum*; vvi, *Vitis vinifera*; zma, *Zea mays*. Some of the data is renewed with information from publications including Arabidopsis [24,62], California poppy [46], grape [60], rice [44], and tomato [23,55].

The 48 miRNA families from sweet orange were compared with those from 11 other plant species (Figure 3). Generally, the orange miRNAs had corresponding homolog in at least one plant species; one miRNA miR1061 only has homolog in phylogenetically distant species *Physcomitrella*. Moreover, miR1169 and miR1171 were extraordinary that they only have homolog genes in *Chlamydomonas *which belongs to protista kingdom. These data suggest that some orange miRNAs are ancient. Sweet orange was phylogenetically close to Arabidopsis which have 187 miRNA genes in miRBase v13.0 database; comparison between them showed that 28 miRNA familes were conserved between them. When comparing with the 140 miRNA genes from another fruit crop grapevine, 23 of them were conserved between sweet orange and grapevine (Figure 3).

Putative targets of miRNAs were determined using publicly available algorithms including miRU [56] and WMD3 [57]. Of the 85 known miRNAs, 82 had putative targets, with the exception of miR167a, miR845b, and miR1520c (Additional file 7). Many putative targets are transcription factors and homologs of known miRNA target genes in other plant species, such as SBP for miR156, NAC for miR164, bZIP for miR166, AP2 for miR172 and F-box for miR394. One occasional case in grapevine [60] was also observed in sweet orange: csi-miR535 was predicted to target *squamosa promoter-binding protein-like *(*SPL*) gene while not target to a brassinosteroid signaling regulator as that firstly reported in moss [61].

### Novel miRNAs

After excluding the rRNA, tRNA, snRNA, snoRNAs and known miRNAs, the remained sRNA sequences were used to BLAST search on orange unigenes and 1.2× coverage sequence of sweet orange (from NCBI); and secondary structure prediction were done for each locus using the criteria described by Jones-Rhoades et al. [29]. This analysis revealed that 21 sRNA sequences were perfectly matched ESTs and could be folded into stem-loop structures (Additional file 8). Search of complementary miRNA* sequences showed that 12 of the 21 candidate miRNAs were detected (Table 2 and Additional file 8). Based on the criteria of loci with stem-loop structure producing sequenced mature miRNA and miRNA* species as described in Meyers et al. [51], these 12 sequences were considered as novel miRNAs.

**Table 2:** Potential novel miRNA genes from sweet orange

miRNA ID	Sequences (5'-3')	WT count^a^	MT count^a^	Fold change	Precursor Unigene ID	Start, end	miRNA star
csi-novel-01	UUUUUCGGCAACAUGAUUUCU	2.8	0.0		EY671768	607, 764	Yes
csi-novel-02	UGGAGGCAGCGGUUCAUCGAUC	4.5	3.3	1.4	EY681703	286,417	Yes
csi-novel-03	UAGAUAAAGAUGAGAGAAAAA	220.1	1248.5	5.7	EY683731	19,166	Yes
csi-novel-04	UUCAAGAAAUCUGUGGGAAG	8.9	0.0		EY714194	255, 397	Yes
csi-novel-05	UGAAGGGCCUUUCUAGAGCAC	90.5	29.6	3.1	CN182895	123, 285	Yes
csi-novel-06	UUCCCUAGUCCCCCUAUUCCUA	11.2	1.5	7.5	CX051199	168, 263	Yes
csi-novel-07	GGAAUGUUGUCUGGCUCGAGG	12.3	4.8	2.6	EY672989	141, 318	Yes
csi-novel-08	AGUGGGAGCGUGGGGUAAGAAG	287.1	369.1	1.3	EY676411	137, 282	Yes
csi-novel-09	UUGAGUUCUGCAAGCCGUCGA	0.0	2.7		EY712260	398, 498	Yes
csi-novel-10	AGGUCAUCUUGCAGCUUCAAU	27.9	4.5	6.2	EY739655	272, 634	Yes
csi-novel-11	UGGACAGAGAAAUCACGGUCA	591.5	132.7	4.5	TC19570	105, 191	Yes
csi-novel-12	GCAGCGUCCUCAAGAUUCACA	15.1	4.5	3.4	TC23189	300, 444	Yes

^a ^count was normalized as transcripts per million (TPM).

All the 12 novel miRNAs had a predicted target in at least one orange EST sequence (Additional file 9). Putative targets of four miRNAs were transcription factors, including NAC for csi-novel-01 and csi-novel-11, AUX/IAA TF for csi-novel-03 and YABBY for csi-novel-10. It is notable that one miRNA potentially target disease resistance genes, i.e. csi-novel-06 predicted to target on serine/threonine kinase gene.

**Figure 4:**
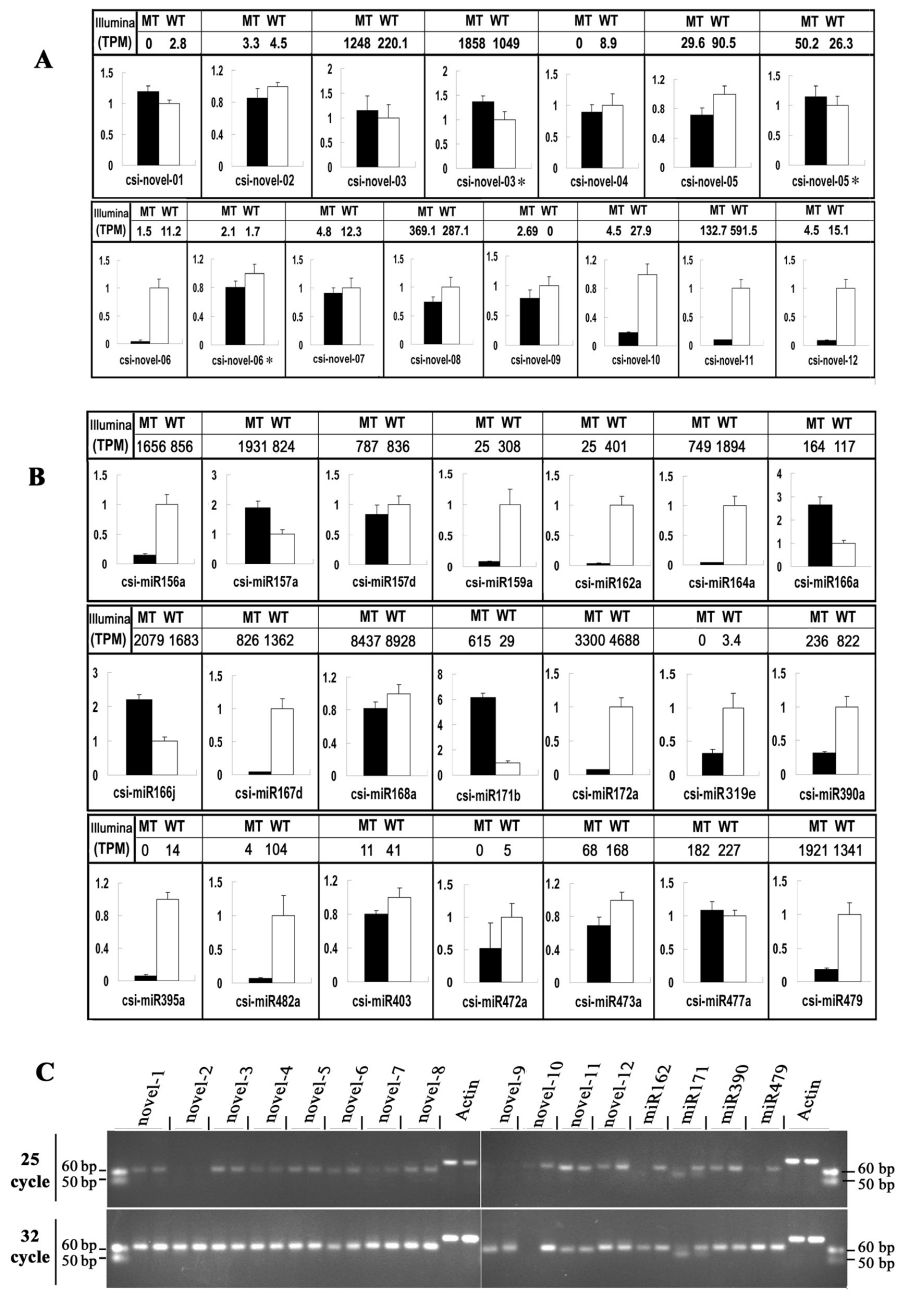
**Expression confirmation of citrus miRNAs derived from high throughput sequencing**. (A). novel miRNAs expression detected by stem-loop qRT-PCR; (B) differentially expressed known miRNAss expression detected by stem-loop qRT-PCR; (C) electrophoresis of the stem-loop PCR products. Each primer has been used for the PCR amplifications on two samples, mutant and wild type.

To confirm the expression of the novel miRNAs, we analyzed the expression of all the 12 novel miRNAs using stem-loop qRT-PCR. All the genes could be detected by qRT-PCR (Figure 4A). Of the 12 novel miRNAs, 8 genes showed similar expression pattern to that revealed from sequencing data of the sRNA libraries. The electrophoresis of the qRT-PCR products confirmed the size and expression of these novel miRNAs (Figure 4C). Moreover, we analyzed the expression of three miRNA*s (csi-novel-03, -05, -06); and results showed that two miRNA*s had different expression pattern from those of corresponding mature miRNAs (Figure 4A).

### miRNA-targets associated small RNAs

To compare the frequency of small RNAs located in miRNA-target genes with other genes, we mined the unique small RNAs (sRNAs) that matched perfectly to miRNA-target genes in sense or antisense strands according to Luo et al. [62]. Target prediction of the 85 known and 12 novel miRNAs produced a total of 555 miRNA-target genes. Then all the 555 miRNA-target genes were searched against the unigenes on which sRNAs were mapped. This analysis in WT showed that 13,811 sRNAs in sense and 8,281 sRNAs in antisense were mapped perfectly on orange unigenes (Table 1; Additional file 1). Of the mapped unigenes in WT, 76 were miRNA-target genes: 21 of them produced sRNAs in sense strand, 11 in antisense and 44 in both sense and antisense (Table 3). For MT sample, 18,142 and 10,651 sRNAs were mapped in sense and antisense, respectively (Table 1; Additional file 2). Similar analysis revealed that 97 miRNA-target genes match sRNAs perfectly, with 20 in sense strand, 27 in antisense and 50 in both strand (Table 3; detail in Additional file 10). This study showed that 13.7% and 17.5% of the miRNA-target genes in WT and MT could be the precursors of sRNAs. The high frequency of miRNA-targets associated sRNAs suggests that miRNA-target genes are hotspots for generation of sRNAs.

**Table 3:** Summary of small RNAs generated from miRNA-target genes

	Producing sense small RNAs	Producing antisense small RNAs	Overlap between sense and antisense	Total sense or antisense
WT miRNA target genes^a^	65 (11.7%)	55 (9.9%)	44 (7.9%)	76 (13.7%)
MT miRNA target genes^a^	70 (12.6%)	77 (13.9%)	50 (9.0%)	97 (17.5%)

^a ^All the 555 miRNA-target genes were used for analysis, detailed gene ID see in Additional file 10.

### Differentially expressed miRNAs between WT and MT

The frequency of each miRNAs in the small RNA library could serve as an index for the estimation of the relative expression abundance of miRNAs. To investigate the expression change of miRNAs between WT and MT, the abundance of each miRNA was normalized as transcripts per million (TPM). A total of 51 known miRNA genes and 9 novel miRNAs were up- or down-regulated significantly at 0.01 level based on Z-score statistics (Table 2 and Additional file 5). Among these 60 differentially expressed genes, 52 miRNAs showed a two-fold or greater (ratio > 2 or < 0.5) expression difference between MT and WT. Table 4 summarized the 20 miRNAs with the most significant expression changes between WT and MT.

**Table 4:** Top 20 differentially expressed miRNAs between WT and MT

miRNA	Sequence	WT count^a^	MT count^a^	Fold change	p-value
**Higher expression in MT**					
csi-miR479	UGUGAUAUUGGUUCGGCUCAUC	1341.6	1921.5	1.9	0
csi-miR156a	UGACAGAAGAGAGUGAGCAC	856.2	1656.1	2.6	0
csi-miR167a	UGAAGCUGCCAGCAUGAUCUA	759.6	1967.3	21.6	0
csi-miR171b	CGAGCCGAAUCAAUAUCACUC	28.5	615.4	4.5	0
csi-novel-08	UAGAUAAAGAUGAGAGAAAAA	220.1	1248.5	1.5	0
csi-miR166j	UCUCGGACCAGGCUUCAUUCC	1682.9	2078.5	3.1	2.85E-22
**Lower expression in MT**					
csi-miR482a	UCUUCCCUAUGCCUCCCAUUCC	103.9	4.2	15.6	0
csi-miR162a	UCGAUAAACCUCUGCAUCCAG	401.0	25.7	12.4	0
csi-miR159a	UUUGGAUUGAAGGGAGCUCUA	307.8	24.8	3.5	0
csi-miR390a	AAGCUCAGGAGGGAUAGCGCC	822.2	235.8	2.5	0
csi-miR164a	UGGAGAAGCAGGGCACGUGCA	1894.0	749.0	1.6	0
csi-miR167d	UGAAGCUGCCAGCAUGAUCUGA	1362.3	825.8	1.4	0
csi-miR172a	AGAAUCUUGAUGAUGCUGCAU	4688.4	3299.7	1.4	0
csi-novel-11	UGGACAGAGAAAUCACGGUCA	591.5	132.7	5.7	0
csi-miR156b	CUGACAGAAGACAGUGAGCAC	41.3	1.8	2.5	2.86E-27
csi-miR473a	ACUCUCCCUCAAGGGCUUCGC	168.1	68.1	1.2	2.01E-26
csi-novel-03	UGAAGGGCCUUUCUAGAGCAC	90.5	29.6	8.1	2.87E-20
csi-miR399a	UGCCAAAGGAGAAUUGCCCUG	36.3	4.5	13.8	3.14E-18
csi-miR482c	UCUUGCCCACCCCUCCCAUUCC	29.0	2.1	15.6	8.98E-18
Csi-miR164d	UGGAGAAGCAGGGCACGUGCU	49.1	11.1	4.4	7.6E-17

^a ^count was normalized as transcripts per million (TPM).

To confirm the expression difference of the miRNA, we analyzed the expression of 21 known miRNA genes using stem-loop qRT-PCR in addition to the 12 novel miRNA genes described earlier. The expression level of each gene in the MT and WT was compared with its abundance from the sequencing data of the sRNA library. Results showed that among the 33 miRNAs, 18 known miRNAs and 8 novel miRNAs have the same expression pattern in WT and MT as that from sequencing data (Figure 4B).

### Annotation of potential targets of differentially expressed miRNAs

**Figure 5:**
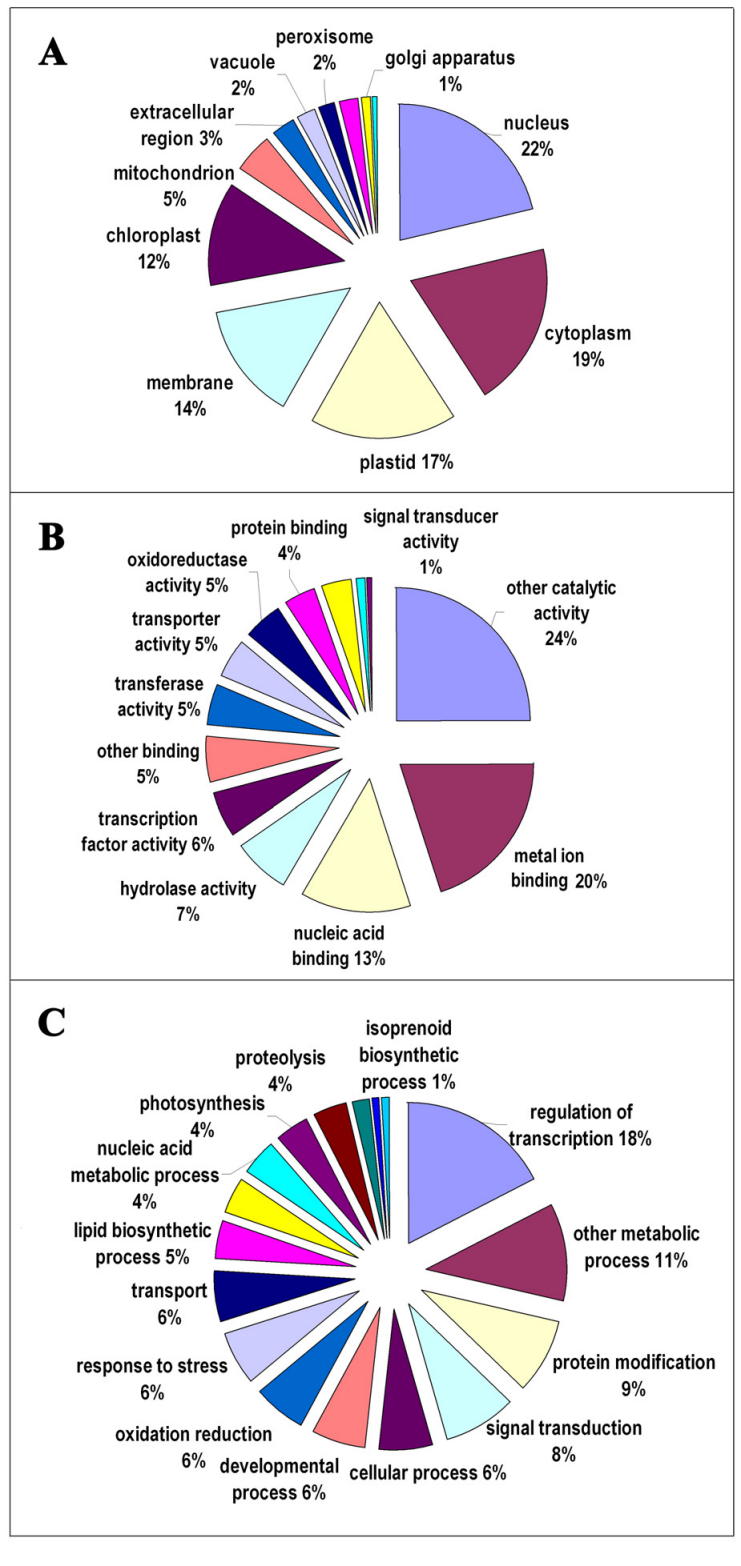
**Gene ontology categories of the predicted target genes of the 60 differential miRNAs**. Categorization of miRNA-target genes was performed according to the cellular component (A), molecular function (B), and biological process (C).

Functional annotations were further performed to investigate which processes are differentially regulated by miRNAs in the mutant. A total of 418 potential target genes were predicted based on the 60 differentially expressed miRNAs in sweet orange (Additional file 11). Interestingly, the potential targets included two important carotenogenic genes, i.e. geranylgeranyl pyrophosphate synthase (GGPS) gene which targeted by csi-miR167 and lycopene β-cyclase (LYCb) gene which targeted by csi-miR1857. To evaluate the potential functions of these miRNA-target genes, gene ontology (GO) categories were assigned to the putative targets of the 60 differentially expressed miRNA genes according to the method described by Morin et al. [63]. Figure 5 summarized the categorization of miRNA-target genes according to the cellular component, molecular function, and biological process. Based on molecular function, the genes were finally classified into 13 categories, as shown in Figure 5B; the most three over-represented GO terms are metal ion binding, nucleic acid binding, and transcription factor activity. Categories based on biological processes revealed that the miRNA-target genes were related to 17 biological processes; the most three frequent terms are regulation of transcription, signal transduction, protein modification (Figure 5C). Moreover, GO representations from this study were compared with that based on all the public unigenes from sweet orange in TIGR gene index database http://compbio.dfci.harvard.edu/tgi/cgi-bin/tgi/GO_browser.pl?species=Orange&gi_dir=csgi; the analysis revealed that the striking differences lies in the high percentage of nucleus, plastid and chloroplast for cellular component, and overpresentation of regulation of transcription, protein modification and photosynthesis for biological process in this study.

The biological interpretation of the target genes of differential miRNAs was further completed using KEGG pathway analysis. A total of 15 different metabolic pathways were found (Table 5), of which some were consistent with biological processes that already revealed by GO analysis. The most represented pathways included carbon fixation (8 enzymes represented), fatty acid metabolism (5), flavonoid biosynthesis (6), terpenoid biosynthesis (3), and ubiquitin mediated proteolysis (3).

**Table 5:** List of the important KEGG pathways more than 3 miRNA-target genes affiliated

KEGG^a ^pathway	Genes^b^	Gene ID	Best E-value
Benzoate degradation	5	EY732960, EY732985, EY748192, TC18796, TC9807	1.00E-125
Carbon fixation	8	EY665527, EY667061, TC11997, TC13950, TC17473, TC24400, EY722705, TC11997	1.00E-128
Ethylbenzene degradation	5	EY732960, EY732985, EY748192, TC18796, TC9807	1.00E-156
Fatty acid metabolism	5	EY732960, EY732985, EY748192, TC18796, TC9807	1.00E-156
Flavonoid biosynthesis	6	EY697662, EY742573, TC14650, TC3846, TC14650, TC3846	6.00E-93
Geraniol degradation	5	EY732960, EY732985, EY748192, TC18796, TC9807	1.00E-156
Glyoxylate and dicarboxylate metabolism	3	EY667061, EY708354, EY722705	2.00E-86
Linolenic acid metabolism	5	EY732960, EY732985, EY748192, TC18796, TC9807	1.00E-122
Methane metabolism	3	EY697129, EY724821, TC20228	1.00E-68
Pentose phosphate pathway	3	TC13950, TC17473, TC15377	5.00E-58
Phenylalanine metabolism	4	EY664657, EY697129, NP13097244, TC20228	1.00E-167
Photosynthesis - antenna proteins	5	EY672715, EY682256, EY701112, EY701374, EY756276	1.00E-102
Ribosome	3	EY681302, EY752996, TC18634	2.00E-91
Terpenoid biosynthesis	3	EY730974, TC363, EY752486	6.00E-84
Ubiquitin mediated proteolysis	3	EY656660, EY665372, EY752038	2.00E-83
Valine leucine and isoleucine degradation	6	EY732985, EY732960, EY748192, TC18796, TC9807, TC5927	1.00E-156

^a^KEGG = Kyoto Encyclopedia of Genes and Genomes.

^b^The putative target genes of miRNA genes which differentially expressed significant at 0.05 level between the mutant and wild type

**Figure 6:**
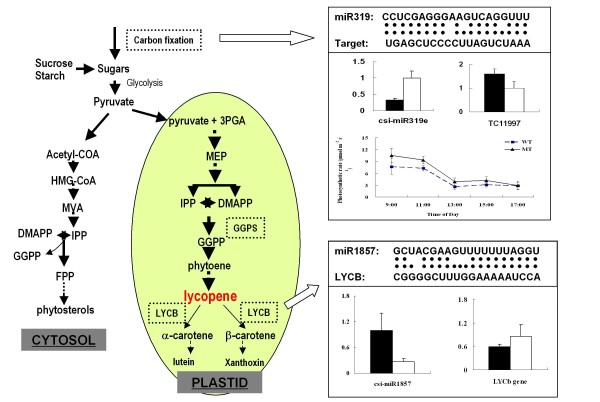
**The model for the biological processes possibly regulated by miRNAs in the red-flesh mutant of sweet orange**. The pathway was modified based on Xu et al. (2009) [13]. The two biological processes, the carotenogenesis and photosynthesis, were high ranked processes that differential miRNA target genes involved in by gene ontology analysis. The data of photosynthetic rate were extracted from our previous in situ photosynthesis analysis [13]. The expression profiles of miRNA319 and miR1857 are complementary to the profiles of the corresponding miRNA-target genes (phosphoenolpyruvate carboxylase [TC11997]) and lycopene β-cyclase [LYCb]), implied the possible roles of these miRNAs in regulation the biological processes on posttranscriptional way.

High ranked target genes of the differentially expressed miRNAs were those implicated in three biological processes including regulation of transcription, protein modification and photosynthesis. It is easy to understand the high frequency of terms 'regulation of transcription' and 'protein modification' since miRNA are involved in diverse regulatory events [64]. However, the biological process of photosynthesis needs to be verified. Moreover, two carotenogenic genes were also predicted to be targeted by miRNAs. We further analyzed one gene involved in the carbon fixation (miR319a and putative target phosphoenolpyruvate carboxylase [TC11997]) and another gene involved in carotenoid biosynthesis (miR1857 and putative target lycopene β-cyclase [LYCb]). The results showed that the expression profiles of miRNAs are complementary to the profiles of their target genes (Figure 6), implied the possible roles of miRNAs in regulation of the biological processes that involved in shaping the mutant trait in sweet orange.

## Discussion

We have characterized the small RNA transcriptome in a red-flesh mutant that with abnormal pattern of lycopene accumulation and its wild type. With deep sequencing strategy by Illumina platform, it provides us not only large number of conserved miRNAs but also novel miRNAs. To the author's knowledge, this is the first report of cloning and characterization of large scale sRNAs and identification of novel miRNAs from sweet orange. A total of 5 and 7 million sRNAs were obtained from WT and MT, respectively; and the size distribution of small RNAs (Figure 2) is quite similar to those published from phylogenetically close plant species such as Arabidopsis [65], with the total percentage of 21-24 nt sRNAs being more than 90% and percentage of 24 nt sRNAs over 50%. This suggests that the Illumina sequencing data of sRNA libraries was reliable.

### miRNAome in sweet orange

In this study, 85 conserved miRNAs belonging 48 families as well as 12 novel miRNA families were identified in sweet orange. The miRNAs cloned in this study were compared with those from previously reported citrus miRNAs [36,66,67], of them four sweet orange miRNAs were not overlap with this study. These results suggest that at least 101 miRNAs exist in the miRNA transcriptome (miRNAome) of sweet orange. We believe that this is a conservative estimate since only fruit samples were used in this study. The miRNA species in wild type and mutant was different, i.e. 69 miRNAs were detected in WT sample while 66 miRNAs were in MT sample. Moreover, the expression levels of each miRNA varied greatly within the transcriptome, i.e. in MT the abundance ranged from 0.9 TPM for csi-miR1858 to 8437 TPM for csi-miR168. All these data implied that miRNAome, as small RNA transcriptome [38], is distinct and complex though miRNA sequences are believed to be conserved across different plant families [23,29,68].

Take a broader view on the small RNA transcriptome of sweet orange, we noticed that the size distribution pattern of sRNAs from sweet orange (the total percentage of 21-24 nt sRNAs > 90% with 24 nt sRNAs > 50%) is quite similar to Arabidopsis as described earlier [65]; however, the distribution pattern is strikingly different from that in *Pinus contorta*, a conifer species [37] where 24-nt RNAs was less frequently (2.5%) and 21-nt RNAs were more abundant (> 50%). The striking difference also existed when compare the sweet orange small RNA transcriptome with another gymnosperm plant species, Chinese yew (*Taxus chinensis*) [50]. When compared with monocot species rice, the difference is less severe but still exists, i.e. in rice 24-nt sRNAs has the highest percentage (approximately 20%) followed by the 21-nt sRNAs (approximately 10%) [37]. These analyses indicated that the small RNA transcriptome is complicated across plant species and can be significantly different between phylogenetically-distant plant families.

A total of 12 novel miRNAs were identified from sweet orange, as evidenced from the biogenesis characteristics of miRNAs including the stem-loop structure of the pre-miRNAs (Additional file 8) and sequencing of the miRNA* in the libraries. The expression of all the 12 miRNAs was also detected by qRT-PCR analyses. Additional qRT-PCR analyses of miRNA*s revealed that some miRNA* species can accumulated at certain levels in fruits; moreover, the expression pattern of miRNA*s in WT and MT fruits can be different from that of miRNAs. For example, csi-novel-05 has lower expression in MT (30 TPM) than in WT (90 TPM) as confirmed by qRT-PCR analysis; in contrast, csi-novel-05* has higher expression in MT (50 TPM) than in WT (26 TPM) which was also confirmed by qRT-PCR. The differential accumulation of miRNAs and miRNA*s may be due to the different activities of key enzyme in the sRNA-pathway, as shown in rice that the differential expression of AGO genes in different tissues might cause the change of miRNA*s abundance [44].

### miRNA-targets are hot spots for generating sRNAs

The relationship of sRNA genes and the miRNA target genes is one of the hot topics in the phenomenon of "miRNA-associated transitivity" [69-71]. In this study, 13.7% (WT) and 17.5% (MT) of the miRNA putative target genes generated sRNAs. This data implied that miRNA target gene could be hot spots for generating sRNAs. Moreover, high percentage of antisense sRNAs was associated with the miRNA-target genes, with 11.7% and 12.6% for WT and MT, respectively. The antisense sRNAs/transcripts can function in post-transcriptional gene silencing [62,72]. The post-transcriptional gene silencing is mainly caused by formation of dsRNA duplexes; this could be supported from the observation that 75% of the antisense sRNAs have their cognate sense transcripts in WT and 64% happens in MT sample in this study.

### miRNAs in plant fruits

Up to now, miRNA identification in fruits using a cloning approach is rare except in tomato [23] and grape [60]. In this study, cloning from sweet orange fruit led to the identification of 48 conserved miRNA families. Analysis of them revealed that 6 miRNA families are expressed in fruits of the three plant species; but they are not fruit-specific. Furthermore, the comparison between the two fruit crops (85 miRNAs from sweet orange and 140 miRNAs from grapevine) showed that 8 miRNA families are expressed in fruits of both crops, and only miR535 is specific to the two species. These analyses suggested that expression and function of fruit-specific miRNAs across plant species is lacking, and miRNAs involved in fruit ripening may overlap with that function in other organs, or the same miRNAs in different organs may have different functions.

### miRNAs possibly involved in the regulation of lycopene accumulation in the sweet orange red-flesh mutant

The characterization and comparative profiling of entire sets of small RNAs (Small RNA transcriptome), especially the microRNA transcriptome, lays the foundation to unravel the complex miRNA-mediated regulatory networks controlling lycopene accumulation in the red-flesh sweet orange mutant. In this study, a number of miRNAs have been identified to be differentially expressed between 'Anliu' sweet orange (WT) and its red-flesh mutant (MT). Compared with WT, 16 known miRNA genes and one novel miRNA genes were expressed exclusively in MT. On the other hand, we found 19 known miRNAs and two novel miRNAs were WT-specific. Moreover, a total of 51 known miRNAs and 9 novel miRNAs were found to exhibit significant expression changes between MT and WT.

Target prediction of these differential miRNAs could provide information on the biological processes regulated by miRNA. Putative targets were predicted by psRNATarget (the updated version of miRU) [56] and web miRNA designer (WMD3) [57]. This strategy was also used in Arabidopsis for the identification of nutrient-responsive miRNAs and their potential targets [24], though further direct validation is necessary to elucidate the true in-vivo targets. However, the annotations of these potential miRNA target genes did provide an alternate view of gene regulation of the red-flesh trait formation in MT (Additional file 11). We found that three groups of predicted miRNA-target genes are most possibly involved in the mutant trait formation of lycopene accumulation. The first interesting observation is that two potential targets (EY752486 by csi-miR167 and TC5 by csi-miR1857) are the key enzymes of the carotenoid biosynthesis pathway (Additional file 11). They encode geranylgeranyl pyrophosphate synthase (GGPS), a key enzyme for the production of prerequisite molecules for carotenoids accumulation, and lycopene β-cyclase (LYCb), a rate-limiting enzyme in the conversion of lycopene to downstream cyclic carotenes. The expression of LYCb and csi-miR1857 also indicated possible interaction between them because of the complementary expression profile (Figure 6). And we also detected the GGPS gene expression, but failed due to the incorrect sequence information in the current public database. The second interesting target genes are APETALA2 (AP2) transcription factors, including EY726563 by csi-novel-03, and TC7810 by csi-miR172a. In Arabidopsis, one AP2 protein (designated as RAP2.2) has been experimentally validated to interact with phytoene synthase (PSY) gene, which encodes a rate-limiting enzyme for the synthesis of carotenoids. The RAP2.2 gene was also believed to be regulated by miRNA because of the large discrepancy between mRNA and protein levels in the overexpression lines [15]. The data from this study also supported the proposition, and indicated that RAP2.2 (encoded by TC7810 gene in this study) is a potential target of miRNA172a in sweet orange. The third group of target genes is those involved in photosynthesis or carbon fixation (Table 5). The potential target genes associated with photosynthesis fits well with the GO results with large number of genes that are located in chloroplast or plastid. Moreover, our photosynthesis-related gene expression analysis and in situ detection of the photosynthetic rate confirmed that photosynthesis in MT is stronger than that in WT (Figure 6). The result is quite consistent with our gas chromatographic analyses that the sugars in juice sacs are higher in MT than in WT [11]. Tomato *hp-2 *mutant which manifested with increased carotenoids, primarily lycopene, showed that genes related with photosynthesis are consistently up-regulated throughout fruit ripening [73]. All these evidences implied the important role of photosynthesis in the accumulation of lycopene. Taken together, the annotation of the potential targets of differentially expressed miRNA genes between WT and MT indicated that several miRNA-directed biological processes including carotenoid biosynthesis, transcription regulation, and photosynthesis may be important for the regulation of lycopene accumulation in the sweet orange red-flesh mutant.

## Conclusions

Our study provides a large scale cloning and characterization of miRNAs in sweet orange. The interpretation of the small RNA data uncovered a total of 85 known and 12 novel miRNAs. Meanwhile, comparative profiling of these miRNAs between a spontaneous red-flesh mutant and its wild type revealed that 51 known and 9 novel miRNAs are differentially expressed. Target prediction of the 60 differential miRNAs indicated that two key genes of the carotenoid biosynthesis, geranylgeranyl pyrophosphate synthase (GGPS) and lycopene β-cyclase (LYCb), are under miRNA regulation. Functional annotation of the 418 target genes of differential miRNA revealed that high ranked biological processes are transcription regulation, protein modification and photosynthesis. The expression of miRNAs and target genes involved in photosynthesis and carotenogenesis were further confirmed. Taken together, this study provides new insight into the miRNAome and possible miRNA-directed molecular processes that regulate lycopene accumulation in the red-flesh sweet orange mutant.

## Methods

### Plant materials and RNA preparation

The red-flesh mutant 'Hong Anliu' and the wild type 'Anliu' sweet orange (*Citrus sinensis *L. Osbeck), cultivated at the Institute of Citrus Research located in Guilin, Guangxi Province, China, were used as materials. The mutant 'Hong Anliu' sweet orange (MT) is a spontaneous bud mutation of wild type 'Anliu' sweet orange (WT); and they are with isogenic background as revealed from our previous physiological traits and molecular marker evaluations [11]. Same sampling strategy as previously published [13] was employed. Fruit samples were harvested at 170 days after flowering (DAF) that is the critical stage for the flesh color transition. Three different trees were used, and 10 representative fruits from each tree were collected. All samples were separated into peel and pulp, immediately frozen in liquid nitrogen and kept at -80°C until use. Total RNA was extracted according to Liu et al. [74].

### Small RNA library construction and sequencing

Small RNA library construction and sequencing was performed as described by Yao et al. [49]. Briefly, small RNAs with 16-30 nt in length were isolated from the total RNA of WT and MT samples by size fractionation with 15% Tris-borate-EDTA urea polyacrylamide gel (TBU). After extraction from the gel, sRNA was dephosphorylatd by alkaline phosphatase (New England Biolabs Inc.). Then the small RNAs were ligated sequentially to 5' and 3' RNA/DNA chimeric oligonucleotide adaptors (Illumina). The ligation products were purified from 10% TBE urea polyacrylamide gel. Reverse transcription was performed with the ligated sRNAs as templates. The obtained cDNAs were sequenced using the Illumina 1 G Genome Analyzer by Beijing Genomics Institute (BGI) (Shenzhen, China).

### Small RNA annotation and microRNA identification

Small RNA sequences were extracted from raw reads matching both the last 7 nt of the 5'-adaptor and the first 7 nt of the 3'-adaptor as described by Moxon et al. [23]. After trimming off the adaptors, sequences between 18 and 30 bp in length and reads more than 3 were used to search the Rfam database http://www.sanger.ac.uk/Software/Rfam by BLASTN analysis to discard rRNA, tRNA, snRNA, and snoRNA. The remaining sequences were compared to known plant miRNAs from miRBase v13.0 [75] using BLASTN algorithm to search conserved miRNAs. Sequences in our libraries with identical or with four or fewer mismatches to currently known miRNAs from other plant species were regarded as potential conserved miRNAs. The potential miRNA sequences were used for BLASTN search against sweet orange unigene dataset and 1.2× coverage sequence of sweet orange (from NCBI), the perfectly matched sequences were used for fold-back structure prediction by Mfold program [59].

To identify non-conserved miRNAs from sweet orange, the remained sRNAs were used for BLASTN search against sweet orange ESTs from TIGR gene index database http://compbio.dfci.harvard.edu/tgi/cgi-bin/tgi/GO_browser.pl?species=Orange&gi_dir=csgi and 1.2× coverage sequence of sweet orange (from NCBI), since the sweet orange genome is not fully sequenced. The sequences of perfect match with sRNA sequences were used for fold-back structure prediction by Mfold program [59]. The sRNAs that have stem-loop precursors were regarded as putative non-conserved miRNAs. Furthermore, we searched miRNA* sequences (complementary to miRNA in the precursor molecule) in the sRNA libraries. Only those with miRNA-miRNA* duplexes were regarded as novel miRNAs.

Significance level of the difference of miRNA frequency and abundance between WT and MT was analyzed using Z-score method according to Kal et al. [76]. The significance of differentially expressed miRNAs were set at the *p *value < 0.01.

### miRNA expression confirmation by stem-loop qRT-PCR

The detection of miRNA expression was performed using stem-loop qRT-PCR method according to Chen et al. [53] and Varkonyi-Gasic et al. [54]. Stem-loop primers for reverse transcription and primers for quantitative PCR were list in Additional file 12. Total RNA was reverse-transcribed using SuperScript III Reverse Transcriptase (Invitrogen, US) by a pulse reverse transcription program [54]. Real-time quantitative PCR (qPCR) was performed on the ABI 7500 Real Time System (PE Applied Biosystems, Foster City, CA, USA) using *actin *gene as endogenous control according to Liu et al. [11]. Briefly, the primers for miRNAs and *actin *were diluted in the SYBER GREEN PCR Master Mix (PE Applied Biosystems) and 20 μl of the reaction mix was added to each well. Reactions were performed by an initial incubation at 50°C for 2 min and at 95°C for 1 min, and then cycled at 95°C for 15 s and 60°C for 1 min for 40 cycles. Output data was generated by the instrument on-board software Sequence Detector Version 1.3.1 (PE Applied Biosystems). The electrophoresis of the PCR products was performed on 3% agarose gel for 1 hr under 70 V voltage. The marker contained two fragments, 50 and 60 bp in length, the sequences information was: GTCGTATCCAGTGCAGGGTCCGAGGTATTCGCACTGGATACGACCCGATG and CGGCGAAGATTGAAGTGAAGGTGCAGGGTCCGAGGTATTCGCACTGGATACGACTTCCGA respectively.

### Target prediction of miRNAs

Target prediction of miRNAs from sweet orange was performed based on methods described by Pant et al. [24]. Two publicly available algorithms, miRU [56] and WMD3 [57] were used in the prediction. The sweet orange EST sequences from TIGR database as described earlier were used to predict the targets. The hybridization energy of each miRNA-predicted target duplex (ΔG) was determined from WMD3 output.

### Functional assignments of the potential targets of differentially expressed miRNAs

To investigate biological processes possibly regulated by miRNAs, assignments of putative functions to potential target genes of differentially expressed miRNAs were performed using annot8r program, which was run locally to BLAST against a reference database that stores UniProt entries, their associated Gene Ontology (GO), Enzyme Commission (EC) and Kyoto Encyclopaedia of Genes and Genomes (KEGG) annotations [77]. The GO categorization results were expressed as three independent hierarchies for biological process, cellular component, and molecular function [78]. The biological interpretation of the potential targets was further completed by assigning to metabolic pathways using KEGG [79].

### The expression detection of two microRNAs target genes

The expression levels of one carotenoid biosynthesis gene (lycopene β-cyclase, LYCb) and one carbon fixation-related gene (phosphoenolpyruvate carboxylase, TC11997) were determined on the ABI 7500 Real Time System (PE Applied Biosystems, Foster City, CA, USA) using *actin *gene as endogenous control according to Liu et al. [11]. The primers are as following: TC11997: forward, GAGATGGTGCAGGCCAAGTT and reverse GTTGAGGAGGTCGCATGGTT; LYCb: forward, GGCTATATGGTGGCAAGGACTT and reverse, CAGAATTGAGGCTTCGAACGA. The data of in situ rates of photosynthesis in the mutant and the wildtype were extracted from our previous data [13].

## Data access

The small RNA sequence data from this study have been submitted to Gene Expression Omnibus (GEO) under accession No. GSE18207 at website: http://www.ncbi.nlm.nih.gov/geo/query/acc.cgi?token=bvcnfememqckehi&acc=GSE18207

## Abbreviations

AGO: ARGONAUTE; AP2: APETEAL2; bZIP: Basic zipper; DAF: Days after flowering; GO: Gene ontology; KEGG: Kyoto Encyclopedia of Genes and Genomes; miRNA: microRNA; NAC: NAM, ATAF, and CUC; PEPC: phosphoenolpyruvate carboxylase; rRNA: Ribosomal RNA; Rubisco: Ribulose-1,5-bisphosphate carboxylase oxygenase; SBP: Squamosa promoter-binding protein; snRNA: Small nuclear RNA; snoRNA: Small nucleolar RNA; sRNA: small RNA; siRNA: short interfering RNA; TPM: Transcripts per million; tRNA: transfer RNA.

## Authors' contributions

QX, YLL, ADZ and KQY are responsible for generating the sRNA data and for interpretation of the results. YLL and JLY carried out qRT-PCR experiments. QX drafted the manuscript. XMW and WWG participated in research design and statistical analyses. XXD proposed and supervised the research. All authors read and approved the final manuscript.

## Supplementary Material

Additional file 1 **WT-derived sRNAs from coding RNA**. This file contains the information of WT-derived sRNAs which were mapped on protein-coding EST genes from sweet orange (*Citrus sinensis*). Click here for file

Additional file 2 **MT-derived sRNAs from coding RNA**. This file contains the information of MT-derived sRNAs which were mapped on protein-coding EST genes from sweet orange (*Citrus sinensis*). Click here for file

Additional file 3 **WT-derived sRNAs from non-coding RNA**. The file lists WT-derived sRNAs which were mapped on non-protein coding RNAs (tRNA, snRNA, snoRNA and rRNA). Click here for file

Additional file 4 **MT-derived sRNAs from non-coding RNA**. The file lists MT-derived sRNAs which were mapped on non-protein coding RNAs (tRNA, snRNA, snoRNA and rRNA). Click here for file

Additional file 5 **Known citrus miRNAs**. Known citrus miRNAs and their expression in WT and MT sweet oranges. Click here for file

Additional file 6 **Fold-back structures for known miRNA from sweet orange (*Citrus sinensis*)**. Precursor sequences for known miRNAs from sweet orange were shown in black letters with miRNA and miRNA* (The sequence complementary to miRNA in the fold-back structure) sequences highlighted in yellow and pink, respectively. Precursor secondary structures and dG value were produced using the mfold software http://mfold.bioinfo.rpi.edu/. Click here for file

Additional file 7 **Target prediction of citrus known miRNAs**. This file contains the predicted target genes for known miRNA genes in sweet orange. Click here for file

Additional file 8 **Fold-back structures for novel miRNAs from sweet orange (*Citrus sinensis*)**. Precursor sequences for novel miRNA from sweet orange were shown in black letters with miRNA and miRNA* (The sequence complementary to miRNA in the fold-back structure) sequences highlighted in yellow and pink, respectively. Precursor secondary structures and dG value were produced using the mfold software http://mfold.bioinfo.rpi.edu/. Click here for file

Additional file 9 **Target prediction of citrus novel miRNAs**. This file contains the predicted target genes for novel miRNA genes in sweet orange. Click here for file

Additional file 10 **miRNA target genes are hotspots for generation small RNAs**. This file contains the information of citrus sRNA loci located in miRNA-target genes in both sense and antisense way. Click here for file

Additional file 11 **The computational annotation of target genes of differential miRNA genes**. This file shows the annotation information of citrus miRNA-target genes; the annotation was performed using arbidopsis homologous gene and Uniprot database. Click here for file

Additional file 12 **The primer sequence information**. This file lists the primers used for miRNA expression detection by stem-loop qRT-PCR. Click here for file
