# Supplementary material for: Discovery and comparative profiling of microRNAs in a sweet orange red-flesh mutant and its wild type
Source: BMC Genomics. 2010 Apr 17;11:246. doi: 10.1186/1471-2164-11-246 (PMC2864249; doi:10.1186/1471-2164-11-246)

Additional data file 6. Fold-back structures for known miRNA from sweet orange (*Citrus sinensis*).

Precursor sequences for known miRNAs from sweet orange were shown in black letters with miRNA and miRNA* (The sequence complementary to miRNA in the fold-back structure) sequences highlighted in yellow and pink, respectively. Precursor secondary structures and dG value were produced using the mfold software (http://mfold.bioinfo.rpi.edu/). The sequence of secondary structure herein is the same as or a part of the precursor sequences.

MIR156a dG = -49.30 kcal/mol CX663821

CGAGAGAGCTACTGACAGAAGAGAGTGAGCACACGCAGGTAATTGTATTAGAAAATATCTTTGCAGGTGCGTGCTCGCTCCTCTTC TGTCAGCGTCATTTTTG

```
          10        20         30        40        50
C-|   GA  UA            -         A   AG    UU    A
  GAGA  GC  CUGACAGAAGAG AGUGAGCAC CGC  GUAA  GUAUU G
  UUUU  UG  GACUGUCUUCUC UCGCUCGUG GUG  CGUU  UAUAA A
GU^   AC  C-            C         C   GA    UC    A
 100        90        80        70        60
```

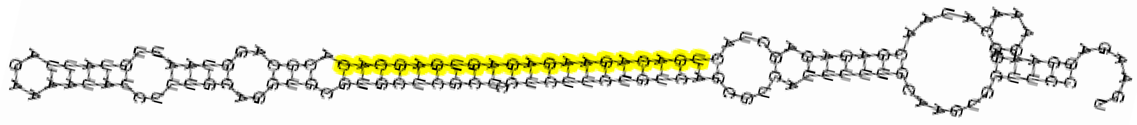

MIR156c dG = -24.90 kcal/mol BUZY276753.g1

ATAAAGATGATGATGACAATGAATTTTGTAGAGAAAGAGAAGAGAGCACAAACTTTTTGCTGAAAGTAGCTTTGATTCGATGTGTA
TCGGTTCATAGATAATGAGTTTTCAAGTCTATTTTAATAGAATACTAAAAGTTAGCTCTAAAAATC

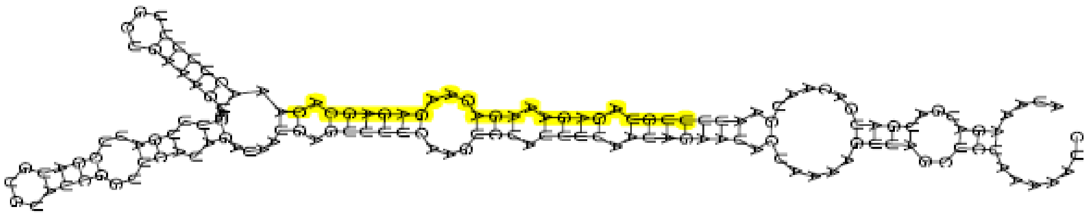

MIR159a dG = -79.40 kcal/mol EY675076:299:514:+ 216(nt)

GGGTTAAGGAGTGGAGCTCCTTGAAGTCCAAAAGAGGTTCTAGCAAGGGTAAATTGAGCTGCTGAGCTATGGATCCCACAGTCCTA
TGTATCAGATTTTAGGGTTTGTTTCTGATGATGGTGATGATAGGCTTGTGGCTTGCATATTTCTGGACCTTCATTACCCCTTAAGTTTT
TACCCCTTTTTTTGGATTGAAGGGAGCTCTACCTCTCTACC

```
         10          20          30            40          50          60          70          80          90           100
G     UA   GA              GA              ---     UCU    --      UA    U    CUG   -    C      GAUC       U      G       GAUU--|       UU
 GGU    AG   GUGGAGCUCCUU   AGUCCAAAAGA    GGU     AGC   AAGGG   AAU GAG    CU GAG UAUG     CCACAG CCUAU UAUCA        UUAGGG   \
 CCA    UC   CAUCUCGAGGGA   UUAGGUUUUUU    CCA     UUG   UUCCC   UUA UUC    GG CUU AUAC     GGUGUU GGAUA GUAGU        AGUCUU   U
-     UC   UC              AG              UCC     UUU    AA      CA    C    CA-   U    U      GUUC       C      -       GGUAGU^       UG
       210         200         190         180         170         160          150         140         130          120         110
```

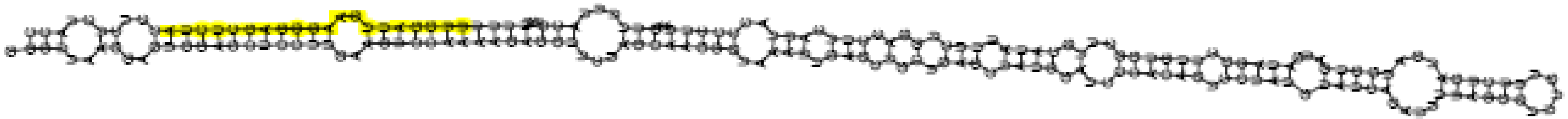

MIR160a dG = -50.10 kcal/mol TC19518:357:454:+ 98(nt)

ATAATTATGTGCCTGGCTCCCTGTATGCCATTTGCAGAGTCAATCGAAACATCGATGGCCTCCGTGGATGGCGTATGAGGAGCCATG
CATATTCCATG

```
              10          20          30           40
AUAAUU          C          CU              UG A  -|    A     A
       AUGUGC UGGCUCC  GUAUGCCAUU  C GA GUCA UCGA A
       UAUACG ACCGAGG  UAUGCGGUAG  G CU CGGU AGCU C
GUACCU          U          AG              GU C  C^    -     A
             90          80          70          60          50
```

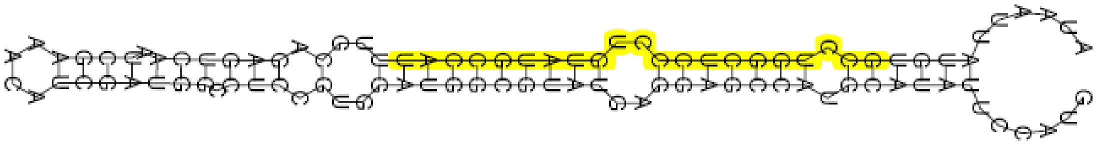

MIR162a dG = -47.30 kcal/mol EY681703:293:407:+ 115(nt)

AGAGTGAAGTCACTGGAGGCAGCGGTTCATCGATCACTTTGTGCAAATTTTGTTGTGAAAAATAACACAAAATACATGAATCGAT
CGATAAACCTCTGCATCCAGCGCTCACTCC

```
          10        20        30        40        50        60
A|      A   CA      G     C     C        AC   UG   CAA        U   GAA
 GAGUGA GU   CUGGA GCAG GGUU AUCGAUC   UU   UG    AUUUUGU GU    A
 CUCACU CG   GACCU CGUC CCAA UAGCUAG   AA   AC    UAAAACA CA    A
C^      -  C-     A     U     A       CU   GU   A--        -  AUA
     110        100       90        80          70
```

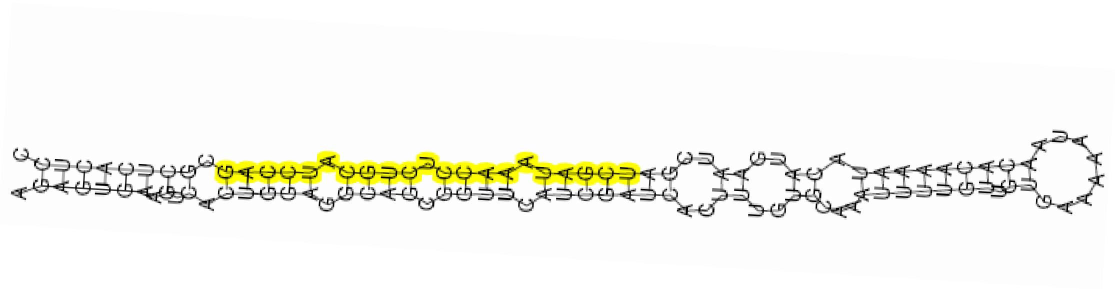

MIR164a dG = -51.10 kcal/mol EY703295:129:316:+ 188(nt) TC23614:965:1152:- 188(nt)

TAGAGCAAGATGGAGAAGCAGGGCACGTGCATTACTAACTCAACCGCACATACATCTACTAACAAAAATTAATTAATCCACACA
TTTTGAAGAAACCAAATCAAGAAGCAGCAAGCTCTTTCTGTTGTTATAGCTCAGTCATGAGTTGAGTTATTTCTTCATGTGCCCTT
CTTCCCCATCATGACCGC

```
          10        20        30        40        50          60          70        80        90
UAGAG   A      A    C             CAUUAC          CGCA    -   AU--    C       .-AAAUUAAUUAAUCCACACAU|    AAG
     CA GAUGG GAAG AGGGCACGUG          UACUCAAC        CAU AC      CUA UAACAA                      UUUG    \
     GU CUACC CUUC UCCCGUGUAC          AUUGAGUUG       GUA UG      GAU AUUGUU                      AAAC    A
CGCCA   A      C    U             UUCUUU          A---    C   ACUC    -       \--------------------^    CAA
        180       170       160       150          140         130                        100
```

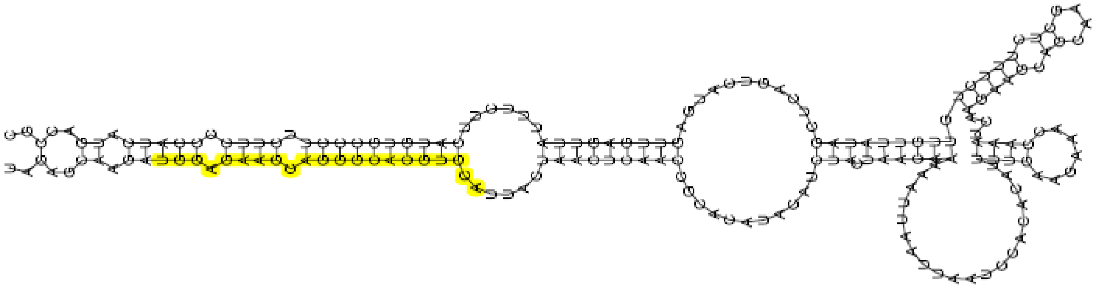

MIR166a dG = -53.80 kcal/mol EY651838:115:274:+ 160(nt) EY672989:149:308:+ 160(nt)

TTTGTTTTGAGGGGAATGTTGTCTGGCTCGAGGACACTGCTTGTTGATCCATTAATTTTACGTATTCTCTCATAGATCTAGCATCTG
AATGGTGGATAACAATATACGGATTCAGCTTATTTGGCGTCGTCGGACCAGGCTTCATTCCCCCCAATATATT

```
         10          20          30           40           50         60           70
UU    U    A           UU         CU     G     .-A     CU       --          A    UUAC    AU--|    CU
   UGU UUG GGGGAAUG    GUCUGG    CGA GAC     CUG    UGUUG    AUCCAUUA UU      GU       UCU    \
   AUA AAC CCCCUUAC    CGGACC    GCU CUG     GGC    AUAAC    UAGGUGGU AG      CG       AGA    C
UU    U    C           UU         AG     G     \ -     AU       AA          A    UCUA    AUCU^    UA
.          150         140        130                  100        90         80
```

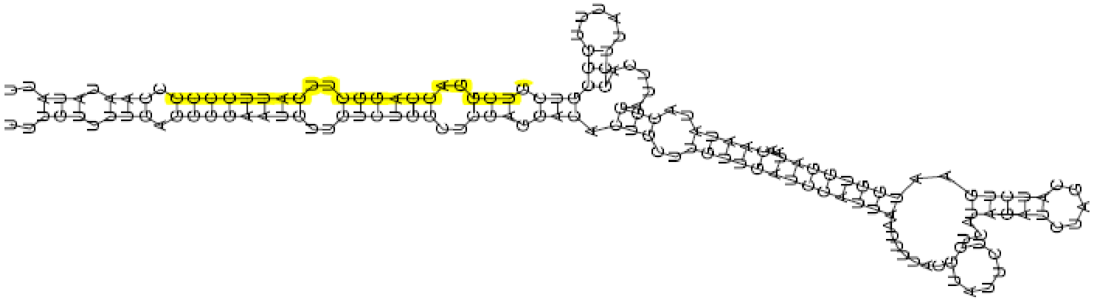

MIR166h   dG = -56.40 kcal/mol   BUZZ101431.b1

TTCCTCCTCGATCATACATGAACGGTTGAGGGGAATGTTGTCTGGTTCGAGATCACTCGTGGACGAATTAATTAATAACTTATTGT
CTTTTGAATGATTTCGGACCAGGCTTCATTCCCCCCAACTCAGTTTAATTGC

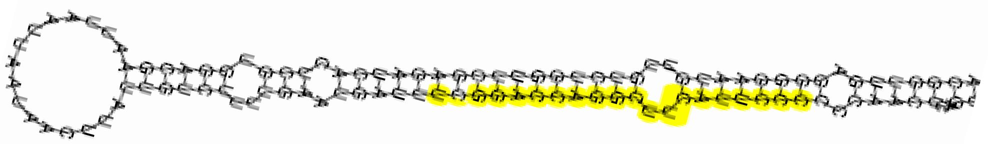

MIR166j dG = -62.10 kcal/mol EY741744:36:179

ATGGTTGATGGGAATGTTGTTTGGCTCGAGGGTCATCTAGGTTTTCAAAATTTTCTCGGACTTTCTTTCTTGCTTTTATTTTTCCAGG
AAAATTATGTTATTTGCCTATGTGATCTCGGACCAGGCTTCATTCCCGTCAACCGA

```
          10        20        30        40          50        60        70
A                UU       CU   G       C     UUU---| A        UC   CUUUCUUUCU
 UGGUUGAUGGGAAUG  GUUUGG  CGA GGUCAU UAGGU       CA AAUUUUC  GGA          U
 GCCAACUGCCCUUAC  CGGACC  GCU CUAGUG AUCCG       GU UUAAAAG  CCU          G
A                UU       AG   -       U     UUUAUU^ A        GA   UUUUAUUUUC
  140       130       120        110       100        90        80
```

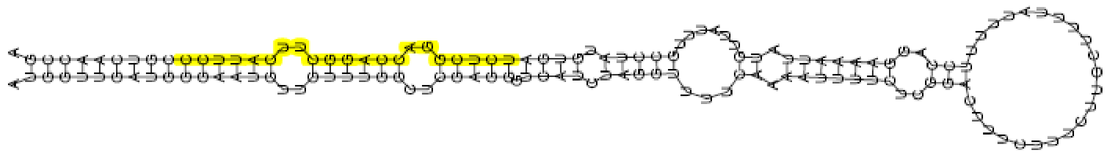

MIR166m   dG = -35.50 kcal/mol   BUZY176722.g1

TTATAATGAAAAAAGGGTATCGGACCAGGCTTCATTCCCTTCAGAATCAAATTCATTATTTGATCTTTTTGAGAGGTTATGGTTGA
TGGGAATGTTGTTTGGCTCGAGGGTCATCTAGGTTTTCAA

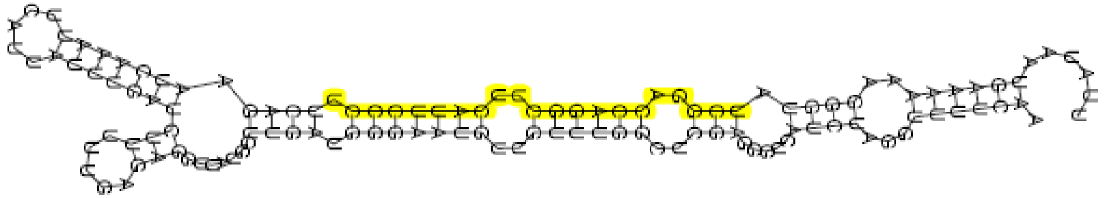

MIR167a dG = -54.50 kcal/mol CX296493

GCACTAGTAGTAGTTGAAGCTGCCAGCATGATCTGAACTTTCCTTGACCTCCATCTCTAGGGAAAGGCCAGATCATCTGGCAGTT
TCACCTATTGATGGTAGC

```
          10        20        30        40         50
 -|  G      U          C       AA       -  CCU
GC ACUA UAGUAG UGAAGCUGCCAG AUGAUCUG   CUUUCCUU GA   C
CG UGGU GUUAUC ACUUUGACGGUC UACUAGAC   GAAAGGGA CU   C
 A^   A     C          -       CG       U  CUA
 100       90        80         70        60
```

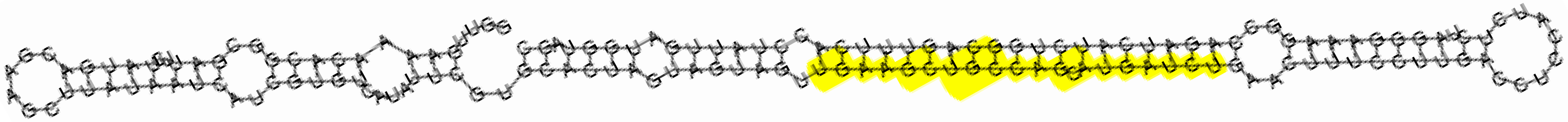

MIR167d dG = -89.90 kcal/mol EY739655:282:622:+ 341(nt)

TTTGAGAGATTGAAGCTGCCAGCATGATCTGGTAATCAACCTTTTTGTATATATATATATATATATTAATTCCTTATAGTTTTTAGATTTAAT
TTCTTTTAATTAGATCCATGGTTTCAATTCTATTGAATAAATGGTGGGGTTTTATATTTTCGTGCAATTATTAAGAGGATAGATGGAATA
GCGCCTTTAAATCCAATCACTTTTTTAGTTTTATTTTGATCTTTTTTGCCCCCTAAAATTAAGGGTAAAGGTTAATATGTGAGAGAGNT
TTAGGGTGTGATTTATTAGCTTCGTAGATGAATGGTTCCATCAGGTCATCTTGCAGCTTCAATTACTCATT

```
           10       20       30       40         50       60       70          80
UU     A           C   C           AAUC    .-UUU     AUAUAUAU    UU      .-UUA|   UUU   UUU
   UGAG GAUUGAAGCUGC AG AUGAUCUGGU     AACC     UUGUAU        AUAUA  AAUUCC     UAGUU   AGA   \
   ACUC UUAACUUCGACG UC UACUGGACUA     UUGG     AACGUG        UAUAU  UUGGGG     AUUAA   UCU   A
UU     A           U   -          CC--    \ ---     CUUU----    U-      \ ---^   UUU   UUA
 .       330       320       310                    150           140        100        90
```

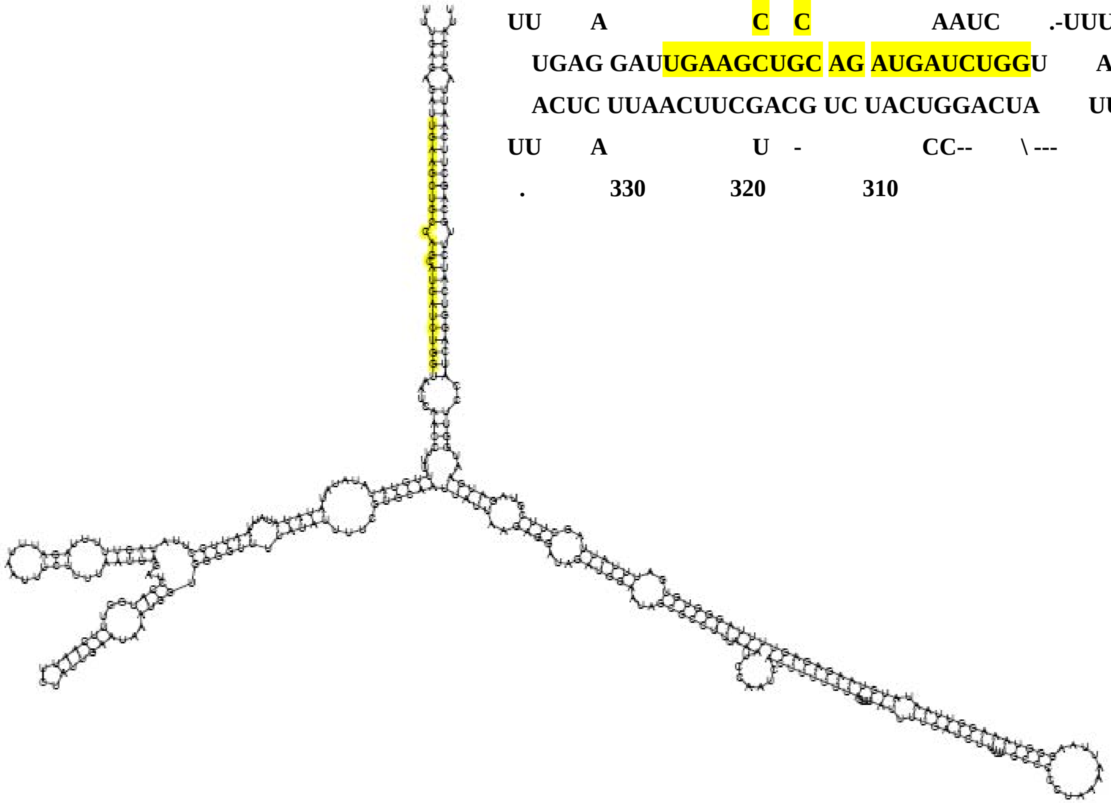

MIR167f   dG = -51.80   kcal/mol   BUZY267574.g1

ATCGGGCACCACTATCAGATGAAGCTGCCAGCATGATCTTAACTTTCCTCCTTTGCTCGAGGAATGATACAGATCATGCGGCAGT
TTCACCTGTTCGTTGGTTGCACGAAATTACGAGTCCAG

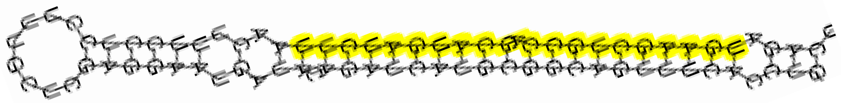

MIR171b dG = -54.80 kcal/mol buzy188852.g1

CAGGCAGAGAAAGCACTTCTTGGCGATTGGAGAAGTAGACATGGTGTGATATTGGTTCGGCTCATCTTAATTAATAGATTCATAT
TTCAGCCCTTAAGGTTCTTACATGTATATATCTAGACGAGCCGAATCAATATCACTCTTGTATGCTTCTTTCTTTTGATT

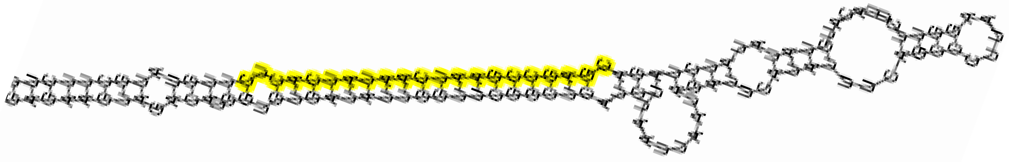

MIR171d dG = -55.10 kcal/mol DN959978

TACTCACGGGATATTGGTGCGGTTCAATAAGAAAACGGTGCTCAATACTTTTTGAGCTCCGCTTTTTGATTGAGCCGCGCCAATAT
CACGTGCCACT

```
         10        20        30        40
UACU-|    G                   A      A   U      UA
     CACG GAUAUUGGUGCGGUUCAAU AGAAA CGG GCUCAA    C
     GUGC CUAUAACCGCGCCGAGUUA UUUUU GCC CGAGUU    U
UCACC^    A                   G      C   U      UU
      90        80        70        60        50
```

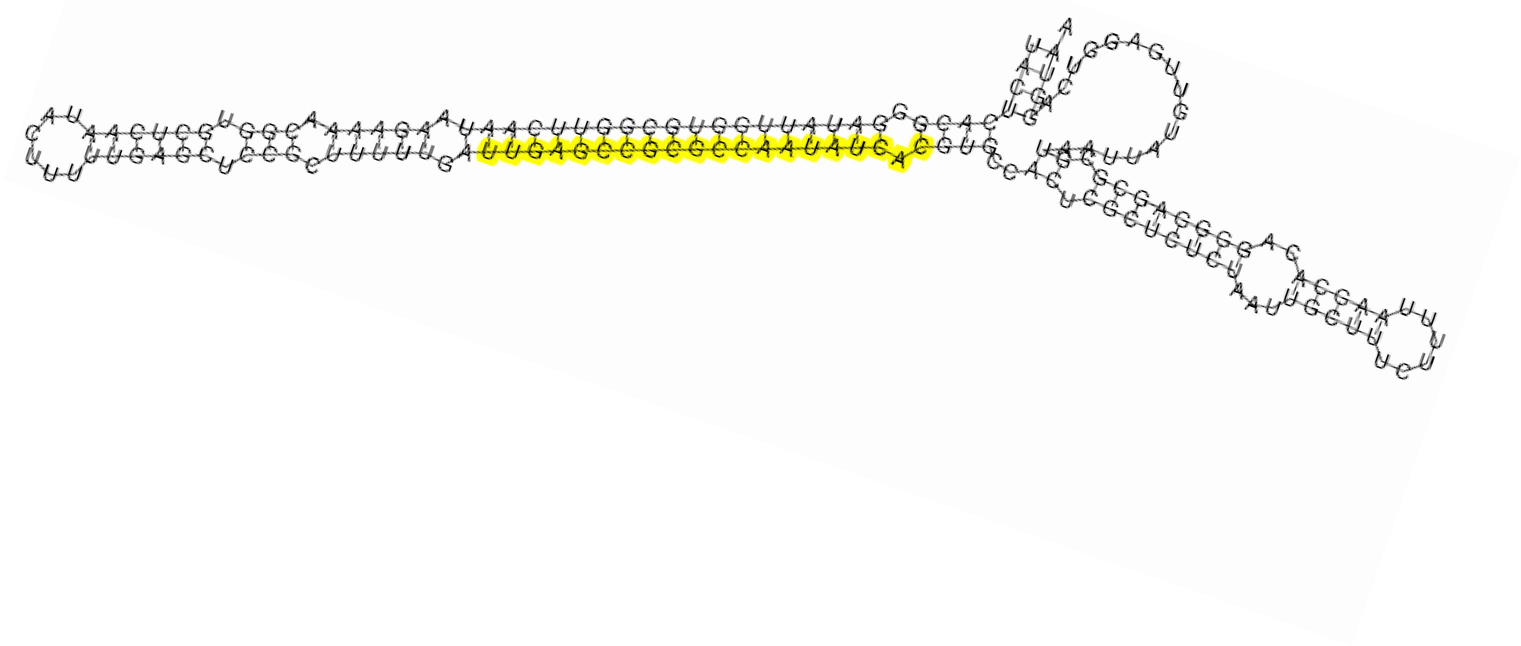

MIR172a dG = -52.20 kcal/mol EY660286:119:245:+ 127(nt)

TTGCTCGCTGTAGCAGCGACGTCAAGATTCACATCCAGTCTAAAGGCAAAAGCAGCAATTTTTCTTCAGTTTGGCTTGCCTGGGTT
TTTGTCAGTGAGAATCTTGATGATGCGGCAACGGCGATGAA

```
             10         20         30           40          50         60
UUGC         A   A    A            A    C--   UCUAA-|    AA    AGC     UUU
    UCGCUGU GC GCG CGUCAAGAUUC CAU   CAG       AGGCA  AGC    AAUU    \
    AGCGGCA CG CGU GUAGUUCUAAG GUG   GUU       UCCGU  UCG    UUGA    C
AAGU         A   G    A            A    ACU   UUUGGG^    --    GU-     CUU
        120        110        100        90        80             70
```

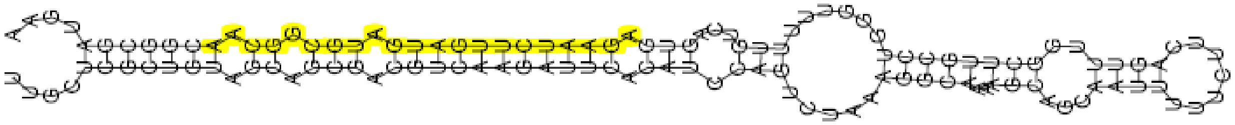

MIR172g dG = -61.10 kcal/mol BUZY19656.g1

ACTGTTTGCAGTTGGAGCACCATCAAGATTCACAAACTATTAGGGTTAGTGAGTGGAGATAATGGTGGCTATTTTATTTTTTTTG
GCCCCTTGCTTCACTTCAAATTGCTCTTTGTTTTGGAATCTTGATGATGCTGCAGCAGCGATAAGTGGCTAAATTATA

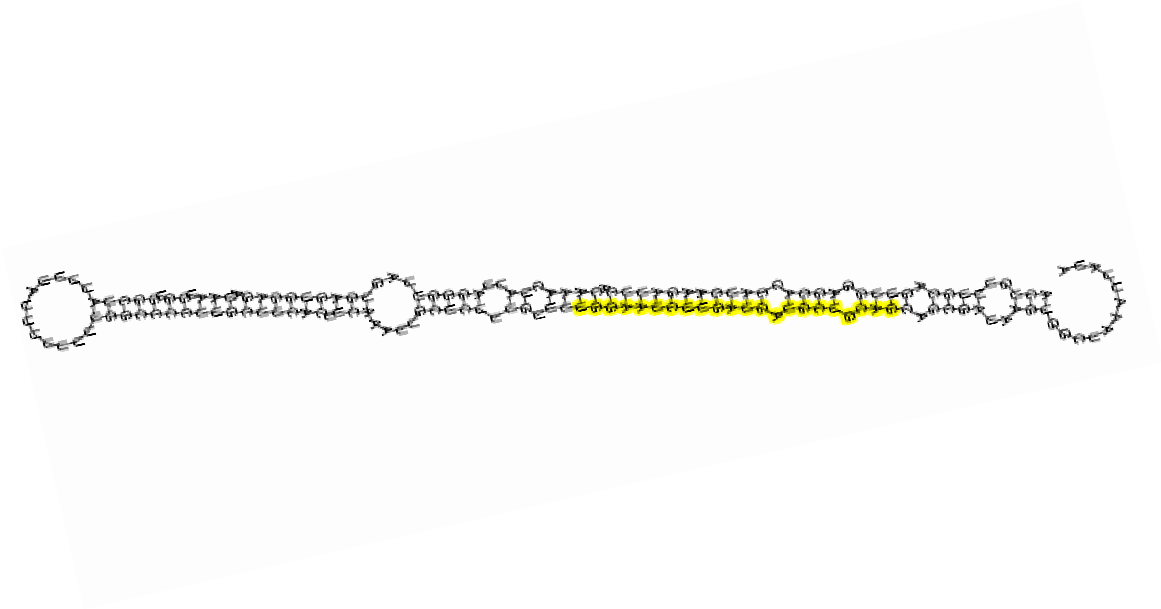

MIR319e **dG = -40.50 kcal/mol** **BUZZ43678.g1**

TCGTCTCTTAATAAAATTGGCGAAAATGGTAGAAATAGGGGTTCCTTGCGGCCCAAAACAGTTGGCTAACTCTGCTCTTCTCTC
TATGTTTTGGACTGAAGGGAGCTCCTAATTCTTCTCTTT

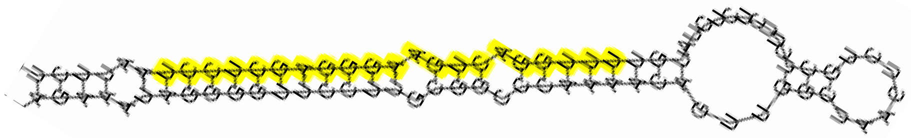

MIR390a dG = -62.60 kcal/mol DR404204:73:209:+ 137(nt)

MIR390a-3 dG = -62.60 kcal/mol DR404204:73:209:+ 137(nt)

AAGTAAAGAAGAATCTGTTAAGCTCAGGAGGGATAGCGCCATGGGTGCCATGAATATGGGAATGATCTAGGGTTTCATGATAAA
TAATGGGCACTGGCGCTATCCATCCTGAGTTTCATGGCTTCTTCTTACTCTCT

```
              10          20          30          40          50          60
A---    A          U     U            G            AUG         AUGAAUAUGGGA|    UCUA
    AGU AAGAAGAA CUGU AAGCUCAGGA GGAUAGCGCC    GGUGCC              AUGA     \
    UCA UUCUUCUU GGUA UUUGAGUCCU CCUAUCGCGG    UCACGG              UACU     G
UCUC    -        C    C           A           ---       GUAAUAAAUAG-^    UUGG
      130         120         110         100            90          80
```

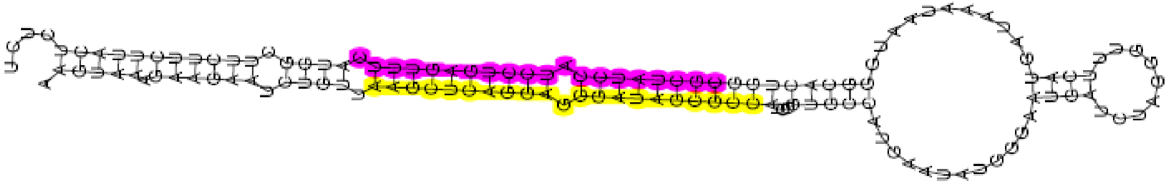

MIR390b   dG = -81.40 kcal/mol   EY733264:396:691:- 296(nt)

AACGCCTACCTACCGTCGATCCCTTCAGAGCGGCGCCCCCGGGGGGGGGGGGGGAGGGCGCGCTGGTGGCCTGGAAGGAATT
GAGTCATTCATTCATTTAGTACTCATCTTTACATACGATACAACAACAACAACTCTTCGAAGTTGAAACATTTTCCTCCTAATTTAT
ACATTAAATTAATGAAACCCCACATTGGAATTTAAGGCCTTCATTGAATTGGATAATTGAGGAAAGATGCGTAAATTATTTTTACTA
TGATTCAAACAGCTCAGGAGGGATAGACAAGGAAGGAGAA

```
          10          20          30           40
AACG    A    ACC    G           A      .-GGCG|      GG
    CCU CCU     GUC AUCCCUUC GAGC            CCCCC   G
    GGA GGA     CAG UAGGGAGG CUCG            GGGGG   G
AAGA    A    A--    A           A      \----^      GG
    290          280          270          50
```

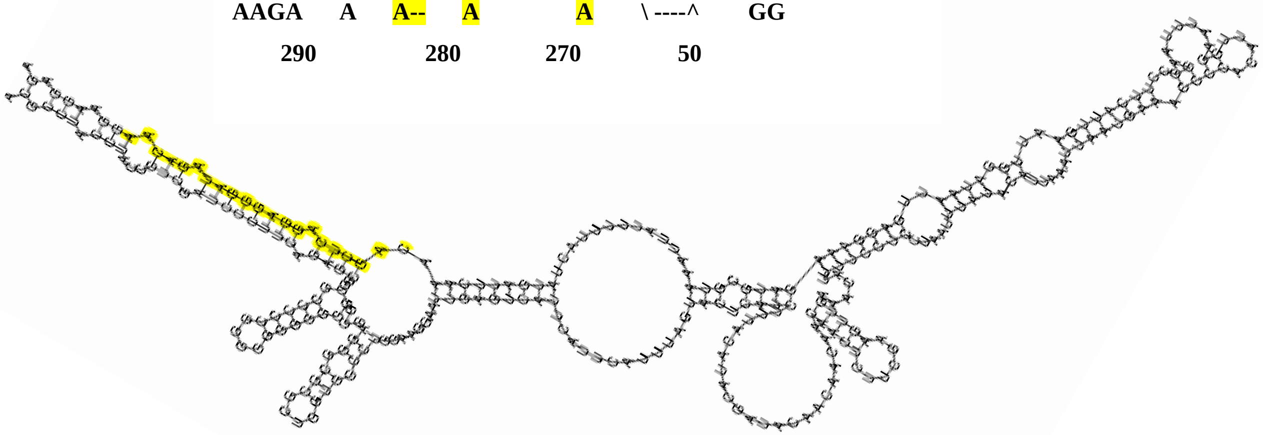

MIR393 dG = -46.30 kcal/mol BUZZ65824.B1

TTCTGCAACTAGAGGATAAATCCAAAGGGATCGCATTGATCCTAAGCCTTAATTATAATTCCCATACTTAATATTCATTAGGACG
ATGCTATCCCTCTGGATTCCTCCTTCGGTAGCTCATATGTATATATATAATCTCTACATA

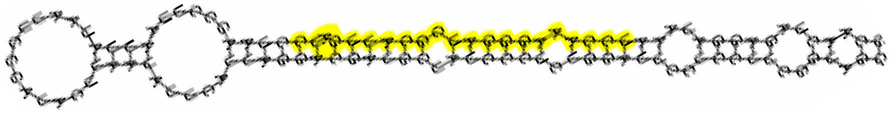

MIR394a dG = -48.80 kcal/mol BUZZ35179.B1

CTTCTTACAGAGTTTATTGGCATTCTGTCCACCTCCGTTCCCCATCGATTGAATCTGAAAGCCTTTTTCTTTTCTTGTTTTTGTGCTT
CTCTCTTTCATGCTCATGGAGGTGGGCATACTGCCAACTGAGCTCTGTTGGCTTCTCTT

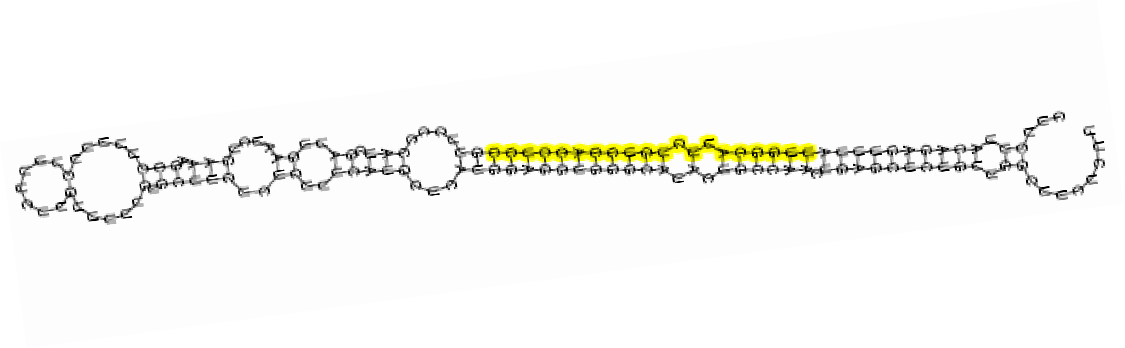

MIR395a dG = -48.10 kcal/mol TC3123:788:888:- 101(nt)

ATGTCTCCGGGAGTTCCTCCGATCACTTCATTGGGGCTATATGATCATTTAGACAAAATTTGTCACCCCACTGAAGTGTTTGGGGGA
ACTCTGGGTGCCAC

```
              10          20          30          40
AU-   CU   G              U         U       .-CUAUA|   U
   GU   CC GGAGUUCCUCCGA CACUUCA UGGGG            UGA C
   CG   GG UCUCAAGGGGGUU GUGAAGU ACCCC            AUU A
CAC   U-   G              U         C       \ -----^   U
 .             90          80          70          50
```

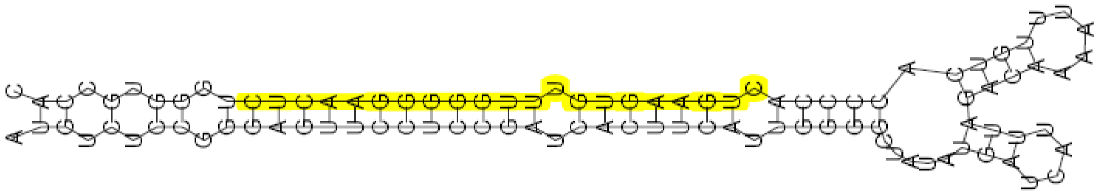

MIR396a   dG = -54.20 kcal/mol   BUZY6224.b1

CGAAGGTCCTCTTTGTGATCTTCCACAGCTTTCTTGAACTGTCTTGAATTTTCCGATCGATACCGGTGCATGATTAATTCCCAACA
CAATTCAAGAAAGCTGTGGAAAACTACTAAGAGGATTTGGTCATCAATCTCTCTTTTGATAGAGATC

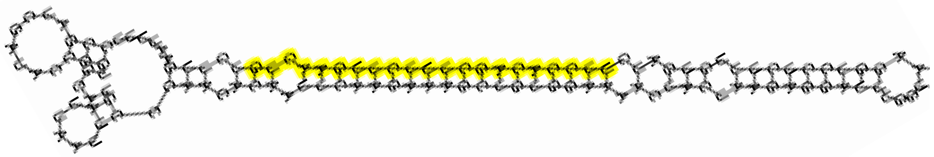

MIR396a dG = -62.50 kcal/mol buzz60759.b1

TATGATCCTCTTTGTATTTTTCCACAGCTTTCTTGAACTGCATCATCTTTGCATGAGGCCACAGACTTGGCATCGCATTTGTTGCGG
TTCAATAAAGCTGTGGGAAGATACAAACAGGTTCAATAAAATTTCAA

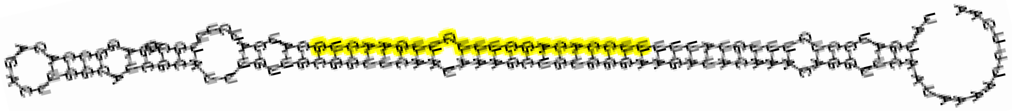

MIR397a   dG = -48.90 kcal/mol   buzz68439.b1

CCAGATTGAAAAAAACATCATTGAGTGCAGCGTTGATGAATATTTGTCGGTGCTTTGCCAATATTTCTCTATACTAGCCAGGATTC
TTTCACCAGCGCTGCACTCGATCATGTTTTTTAGCTCTGCTGGTTCAGGATTATCCAGATAATACGCACA

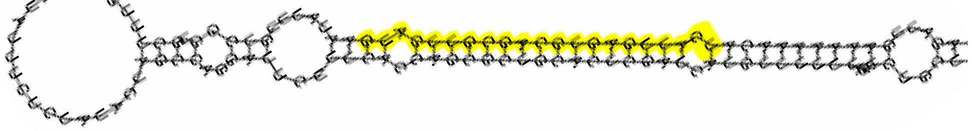

MIR399b dG = -22.90 kcal/mol BUZY234309.g1 (5’)

ATTTGCATCCTTCTGTTTGCCAAAGGAGAATTGCCCTGCCATTCACTCTCGCAGCTAGTTGTAATTGTTGCAAGATTCCAGCAGCC
TCATTCAATTTGTTTGCCAAATTAATGTAACAAACGATTTCATCATTTACCATTCTGAGTTCAATATG

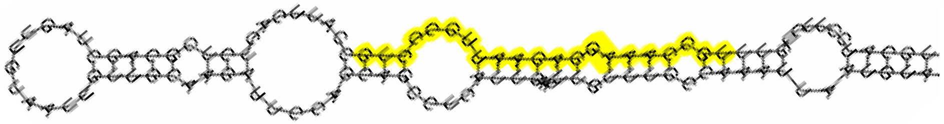

MIR399b dG = -54.20 kcal/mol BUZY234309.g1 (3')

TCAATACAAAGTGACAATGTGGTTGAGGAATAACAGTGCAGTCCTCCTTTGGCGTGCAGATTGCAAAGAGATAATGCATATTATT
TCATTCATTTGCATCCTTCTGTTTGCCAAAGGAGAATTGCCCTGCCATTCACTCTCGCAGCTAG

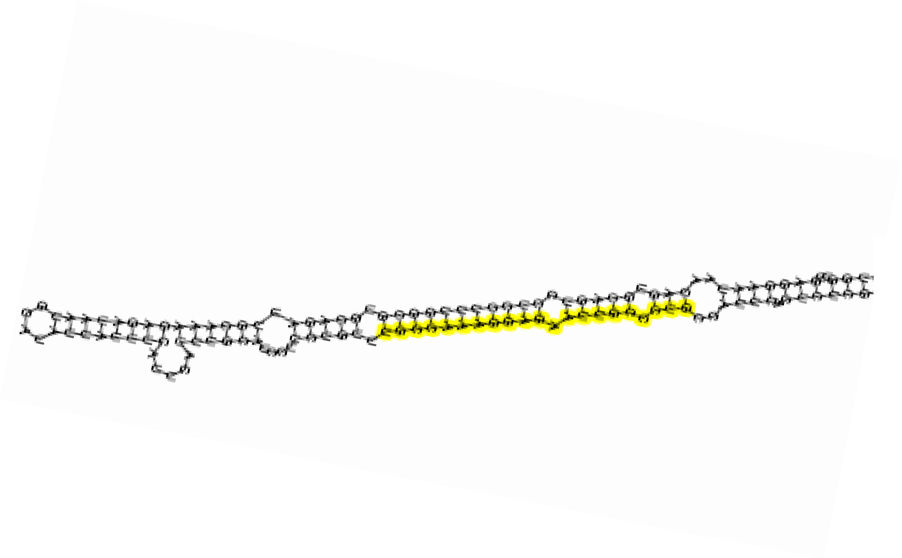

MIR399b dG = -56.50 kcal/mol CV712967:390:519:- 130(nt)

AAAGCAGTTTTAGGGCACCTCTTACTTGGCATGCAATGGTGATTTATTGGTGCAATAATCAGCATTATATTGGTTAATTCGATATCAAT
CCCACTGATATGCCAAAGGAGAGTTGCCCTGTGACTGCTTC

```
          10         20         30           40           50
A          U         C       AC        -   A    U    .-UAU|      AAU
 AAGCAGUU UAGGGCA CUCUU  UUGGCAUG CA UGG GAUU      UGGUGC    A
 UUCGUCAG GUCCCGU GAGAG  AACCGUAU GU ACC CUAA      AUUACG    A
C          U         U       GA        A   C    -    \---^      ACU
.         120        110        100        90               60
```

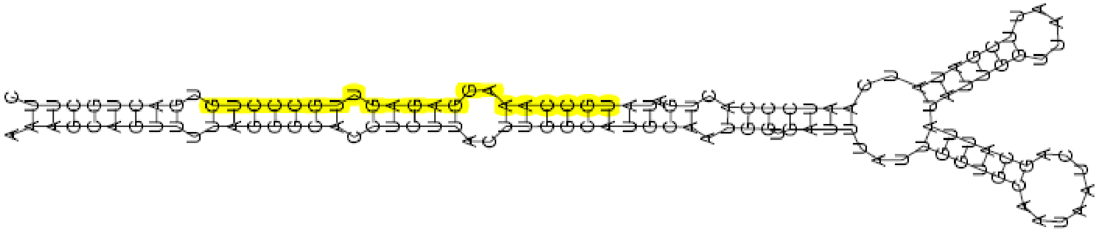

MIR399f dG = -55.20 kcal/mol BUZY206720.b1

CATTGAGGTGCATGCATGCAGTTGCATATTACAGGGCAACATCTCCATTGGCAGGCTGCATACTTATTACATCCATCTCATATATACGTATGTTCTTC
AGTCAGTGTGCCTTCTGCCAAAGGAGATTTGCCCGGCAATTCCTCTGCATTTATTTT

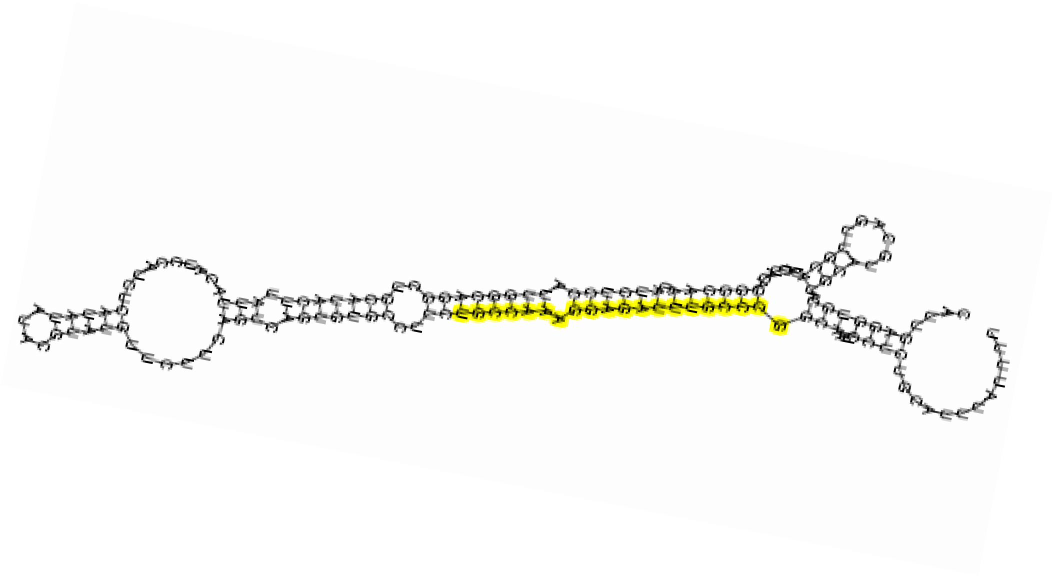

MIR399j dG = -68.50 kcal/mol BUZY89877.b1

GAAGCAGTAAGCGATTTAGTTGTAGGGCTTCTCTCCTTTGGCAGGTGATGGCCAAGGGCAAAGATCATATAATATATATTTATATGGTGCCTATTCTA
ATTATTGTTCTATTTTTTCAATGACCTGCCAAAGGAGAGTTGCCCTATAACTGCTTCAGCTTTTAAATAA

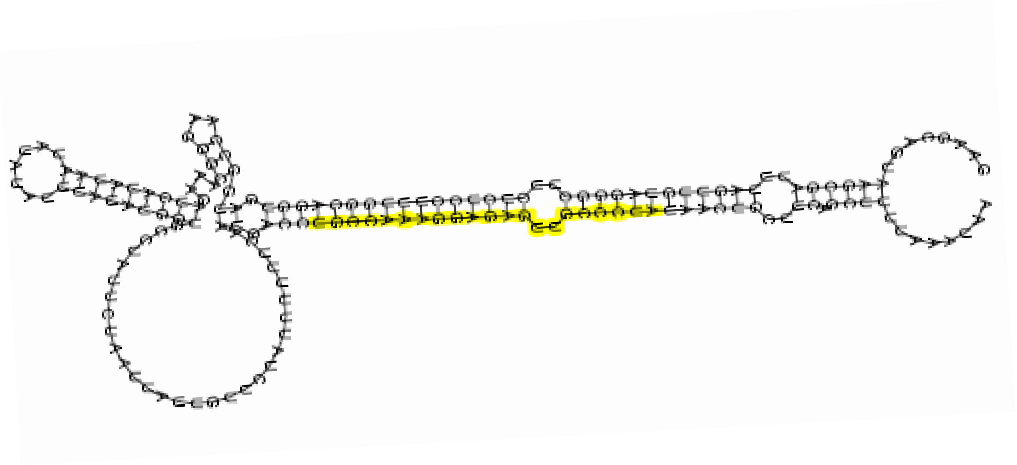

MIR403 dG = -44.60 kcal/mol EY656660:79:208:+ 130(nt)

GAGGCATGTTTCGGGTTTGTGCGTGAATTTAATAAAATAACCGCTTCTTTTAATCTCGTTCCCCCATTTTTTGTTAGTAACAAATTTTT
GCCGTCAGTGTTAGATTCACGCACAAACTCGTGATGTGTCT

```
         10        20        30        40        50        60        70
G|        U                        AAAUA   C   UUCUUUUAAUCUCGUUCCCCCAUUU      A
 AGGCAUGUU CGGGUUUGUGCGUGAAUUUAAUA       AC GC                         UUUGUU \
 UCUGUGUAG GCUCAAACACGCACUUAGAUUGU       UG CG                         AAACAA G
-^        U                        GAC--   C  UUUUU--------------------     U
 .       120       110       100        90                           80
```

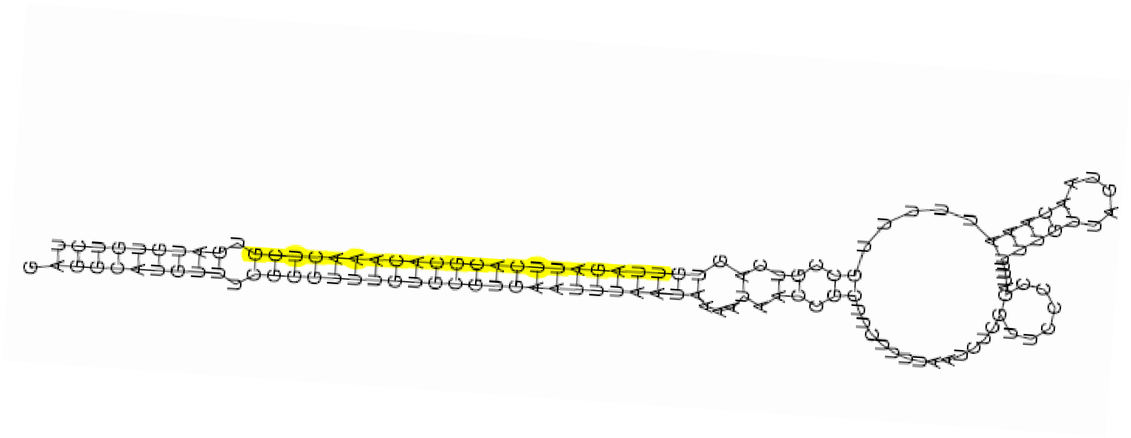

MIR408 dG = -63.10 kcal/mol buzy222719.b1

AGGTAATAATAACTCTTTGGTGTGTGCGTGAGAGAGAGGCAAGAAGCAAGAGACAAAAGACGGGGAACAGGCAGAGCATGGAT
GGAACCATTAACAGGTTCTCTGTTTTGGCTCCTCCCATGCACTGCCTCTTCCCTGGCTCTCTGCCTTCCT

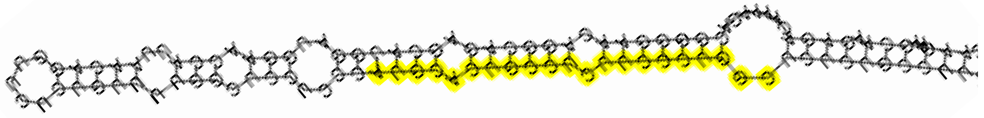

MIR472a dG = -38.80 kcal/mol EY748618:56:149:+ 94(nt)

GGGAAGTCTTGGAGATGGGTGAGTTGGGAAAATAATAACTTTCTTCACGACAGAAAGCAATTTTTTTCCCACACCTCCCATCCCTA
AGATTTTC

```
             10        20          30        40
G|            A       UGA  -        U    AA       UCA
 GGAAGUCUUGG GAUGGG   GU UGGGAAAA AAU  CUUUCU   \
 CUUUUAGAAUC CUACCC   CA ACCCUUUU UUA  GAAAGA   C
-^            C       UC-  C        U    AC       CAG
         90        80          70        60        50
```

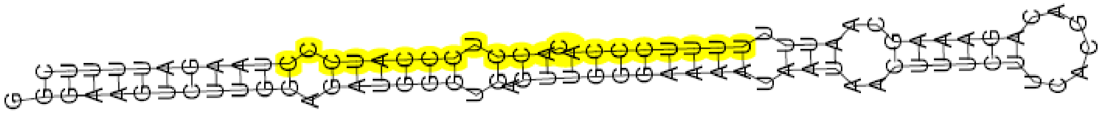

MIR473a dG = -61.40 kcal/mol BUZY181546.g1

CCAAAGCAGTAGAGTCGTTCACTCTCCCTCAAGGGCTTCGCCACAATCCAAATCATGCTGAACAGGTGGGTGGGTTGATGCTGAAGTCCTTGGGGTT
GAGTGATCGACGCCTGTTTTTTTAACTCGCTAATGACGATCGCAGAAGACAACTCGAAGGCTA

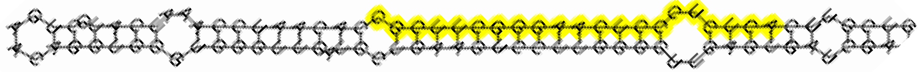

MIR477a　　dG = -62.80 kcal/mol　　BUZY103319.g1

AAAATGGAGAAGCTTCCAACCTCCCTCGAAGGCTTCCAATATACCATGCTTCCTCANATTGGCCTTGTAAGCATGGGAATGTTGGAAACCCTAGGGG
GAGGTTGAAAGCTTATGACTCCATTTAAATCAGTCTGTACGTG

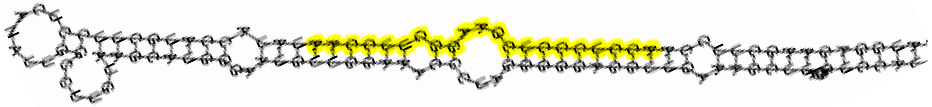

MIR477c dG = -60.70 kcal/mol BUZY181546.g1

CAAAAGCAATCGTCGTTCACTCTCCCTCAAGGGCTTCTCTTCGATCCATATATAACGTACATATGCTCAAATGAGTGGTGATGGATCGACGCTGAAG
CCCTTAGGGCAGAGTGATCGACTCCGGCTTTCTCATTTTCC

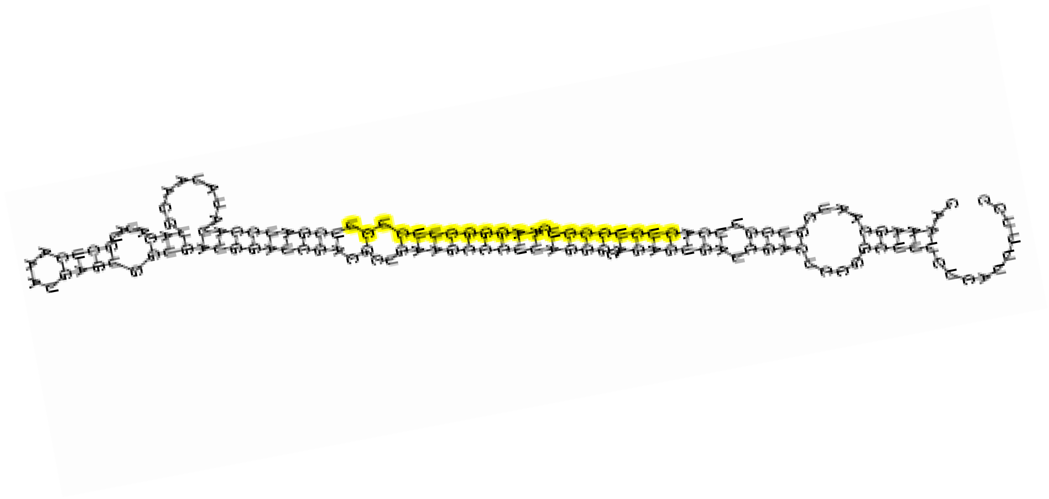

MIR477f **dG = -62.50 kcal/mol** **BUZY103319.g1**

AAATGGAGAAGCTTCCAACCTCCCTCGAAGGCTTCCAATATACCATGCTTCCTCANATTGGCCTTGTAAGCATGGGAATGTTGGAAACCCTAGGGGG
AGGTTGAAAGCTTATGACTCCATTTAAATCAGTCTGTACGTG

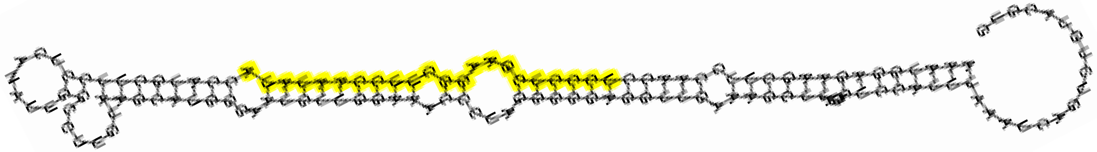

MIR479 dG = -62.50 kcal/mol buzy188852.g1

ATTGGAGAAGTAGACATGGTGTGATATTGGTTCGGCTCATCTTAATTAATAGATTCATATTTCAGCCCTTAAGGTTCTTACATGTATATATCTAGACG
AGCCGAATCAATATCACTCTTGTATGCTTCTTTCTTTTGATTTTGCACC

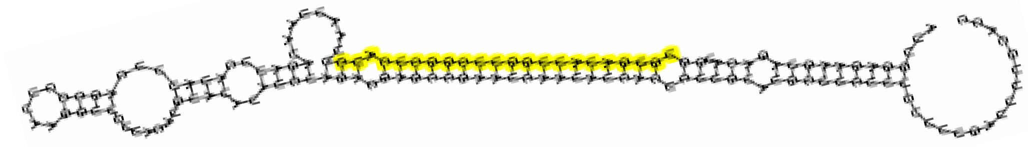

MIR482a dG = -56.30 kcal/mol EY676411:144:272:+ 129(nt)

AGGAAGTTTTGGGAGTGGGAGCGTGGGGTAAGAAGAGAAGAAAGCGTATAATTTTTCTTTTCTTTTTCAATTGAGAAAGAAAATT
ACAAATTCAATTTCTTCCCTATGCCTCCCATTCCTATGATTTCC

```
            10         20          30          40
A          U            -          U       .-A|        CGU
 GGAAGUU UGGGAGUGGGA GCGUGGGG AAGAAG    GAAGAAAG    A
 CCUUUAG AUCCUUACCCU CGUAUCCC UUCUUU    UUUCUUUU    U
-          U            C          -       \ -^        UAA
            120        110         100     60          50
```

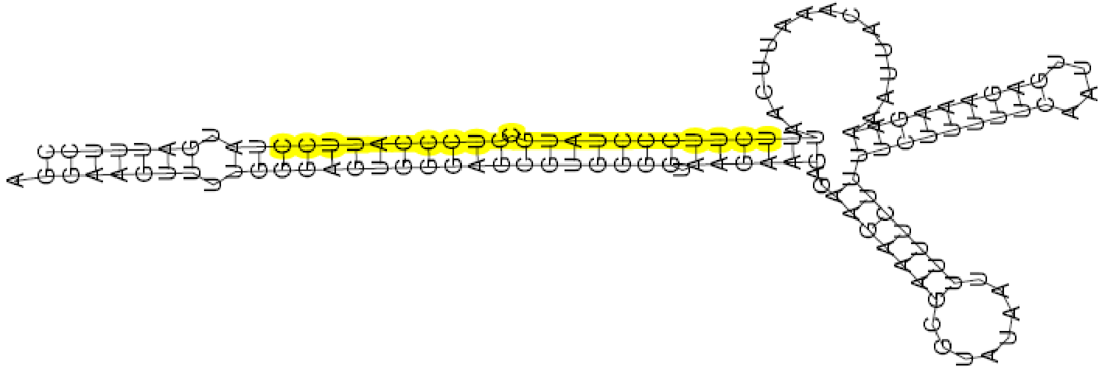

MIR482c dG = -47.50 kcal/mol CK702206:71:179:+ 109(nt)

AGGAAATTTCTGGAATGGGAGGCTTGGCAAGAAGCGATCTATTTTTCTTGTTCAATAAAAAAGAAAAAGAAAATTTTTCTTGCCCA
CCCCTCCCATTCCTTGGATTTCC

```
           10         20          30         40         50
A         CU             CUU-|         CGAUCUA         GUUCA
 GGAAAUUU  GGAAUGGGAGG     GGCAAGAAG          UUUUUCUU      \
 CCUUUAGG  CCUUACCCUCC     CCGUUCUUU          AAAAAGAA      A
-        UU          CCAC^          UUAAAAG          AAAAU
           100        90          80         70         60
```

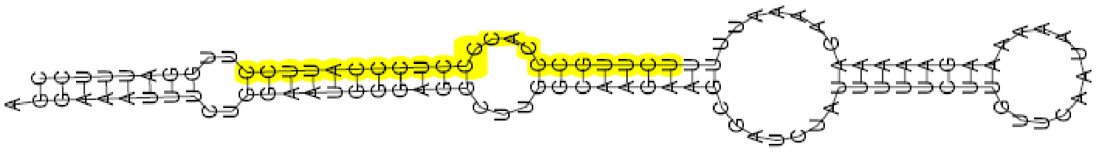

MIR530a (C24170) dG = -48.60 kcal/mol

GGCACGAGATTTTTCTGCATTTGCACCTGCACCTTGCTGTGCTGTTATCTGGGTTGACGAAGATCATCATGATGATCAGCTTCAAAA CTCGAAAGCATGAAGGTGCAGCTGTATATGCAGGTGAATGCC

```
            10        20        30        40        50        60
|   CGAGA   U       U    C         GC      GUUAUC     GAC   ---     C
GGCA     UUU UCUGCAU UGCA CUGCACCUU  UGUGCU      UGGGUU   GAA   GAUCAU A
CCGU     AAG GGACGUA AUGU GACGUGGAA  GUACGA      GCUCAA   CUU   CUAGUA U
^   -----   U       U    C       --    AA----     AA-   CGA     G
            120       110       100              90       80       70
```

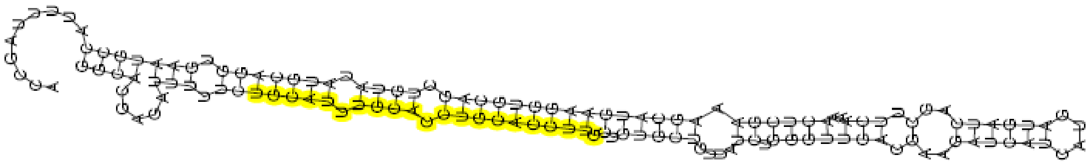

MIR530b dG = -66.60 kcal/mol EY679643:98:251:+ 154(nt)

ATCCTTTATCTGCATTTGCACCTGCATCTTGTTGCGTTATCTGGTTCTGGTTCGTAAAAGCCAAAGCACAAGAAGAGCCAATAGCCG
TCGGCAGATATGGTGGCAGCATGGGGTTATATGCATCAAGGTGCAGTTGCAAGTGCAGATGAATGCC

```
             10         20         30           40           50
AUCC                   C           U      .-UA   -   -|   C       CG
    UUUAUCUGCAUUUGCA CUGCAUCUUG UGCGU     UC UG GUU UGGUU   U
    AAGUAGACGUGAACGU GACGUGGAAC ACGUA     AG AC CGA ACCGA   A
CCGU                   U           U      \ --   A   A^   A       AA
  150        140        130        120         70          60
```

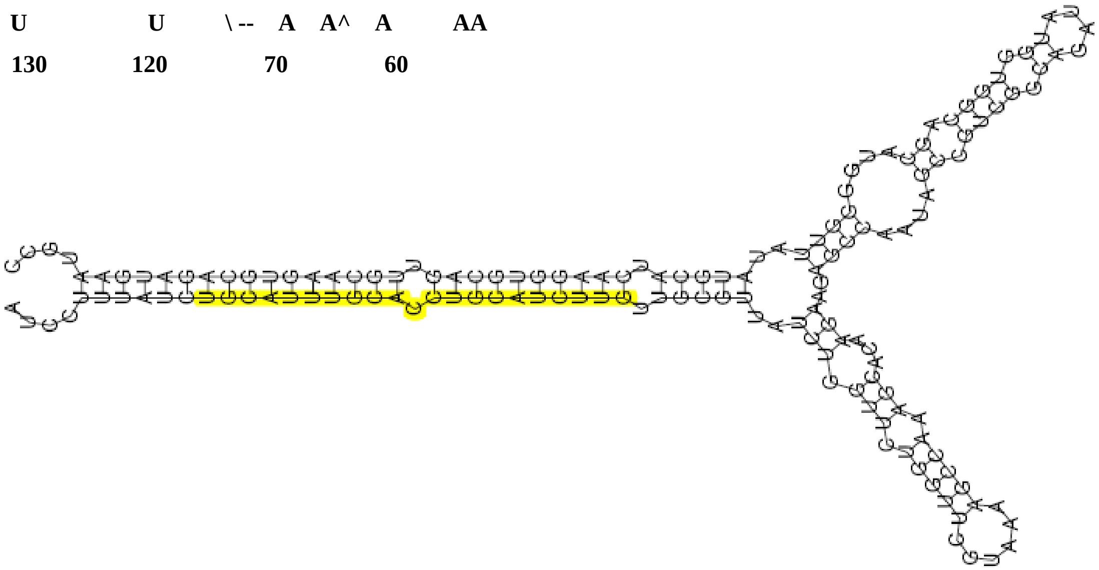

MIR535a dG = -24.40 kcal/mol BUZY211691.b1

ATTTTTTCTAATGAACATTCTTTGTTGCAAGGTTTTCTGATTGTATTGTTACTTTTTTGTGCAGATATATAATCCTTGCCCAAACTC
AAGACAACTTTGTTTGACAATGAGAGAGAGCACACCCGTCAGCATTCGTGCAAA

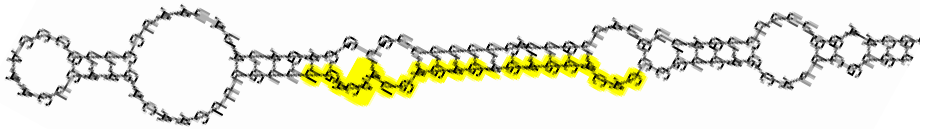

MIR827　　**dG = -30.90 kcal/mol**　　**BUZY251745.g1**

GCTTGTTGATTGTCATCTAATCATTTTCTTTTTCATCCGGGCATTTGAATGATTATATGGTTAGATGACCATCAACAAACATATTCA
TGGTATGGCATGC

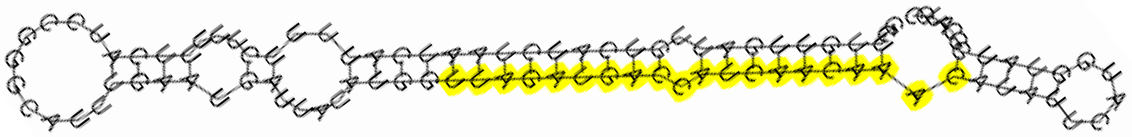

MIR857 dG = -27.20 kcal/mol BUZY105556.G1

ATGTTGAAAATTGATTAAAAAAAAAAAGGTTGAAAAGTCAAATTAGAAGACATAGTTTGAATTCAAATGGGAGGTGGCCGAGA
ACTTTGAAAAGTAATTGGAAGTGGCCAAAACTATTTTGAATGTTGAATGGTGGCTATTGAATGTTGAAA

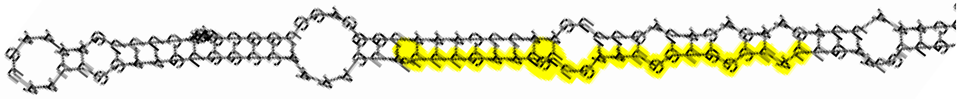

MIR1061   dG = -18.40 kcal/mol   buzy164479.b1

AATTAGATTCAAGGTTTTTAAGTTTTGTTATCACTTCTTACTATTAAAAATAACAAATGATGAGATATGTTATGCTACTGTGAATT
AAATGTACTTTTTAAGACTTAAACTCTTAACTTCTTATTTCAGTAGACGACGTCACATGATTAGAATATTCTTTTAT

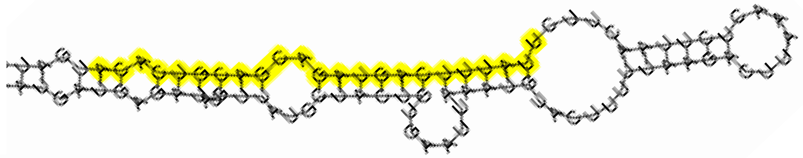

MIR1171 dG = -23.20 kcal/mol buzy271716.b1

AGGAGTGTGGAGTGGGAGTGGGAGTAGGGTGTTTACTTAGACTAAATGAAAGTATGGATTATCAATCAGAATCCTAATTATTTGT
TTACTTTGTCTTGGATTGGGAGTAAATTATTTTAAATTATAATTTTATCCTTATGTACAAAATTATAA

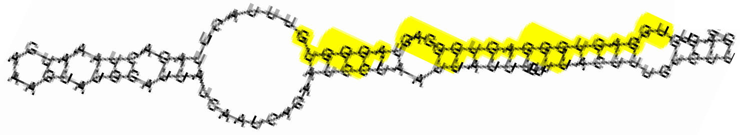

MIR1515 dG = -46.10 kcal/mol EY703412:129:303:+ 175(nt)

TCGATTTGCATCATTTTTGCGTGCAATGATCCCGAATTTGCTCTCTTACTCAACTTTCTTTTTAATTTACAAATATTCATGTTTATTGGT
TTGAAAAATAGGAAAAAAAAATTCTAATTTTTGTTAACGGGGATCTGGATCATTCACGCAAAAATGATTTGACTCGTGAGTTGGC

```
                    10        20        30          40        50        60        70
 -      UG -------              C         .-C      GCUC-|    CUCAAC    UUU      UU    AAU
  UCGAUU  C        AUCAUUUUUGCGUG AAUGAUCC   GAAUUU     UCUUA       UUUC    UUAAU  ACA   A
  GGUUGA  G        UAGUAAAAACGCAC UUACUAGG   CUUAAA     AGGAU       AAAG    GGUUA  UGU   U
 C       GU CUCAGUU             -         \ -      AAAAA^    AA----    UUU      UU    ACU
    170       160       150        140        110       100            90        80
```

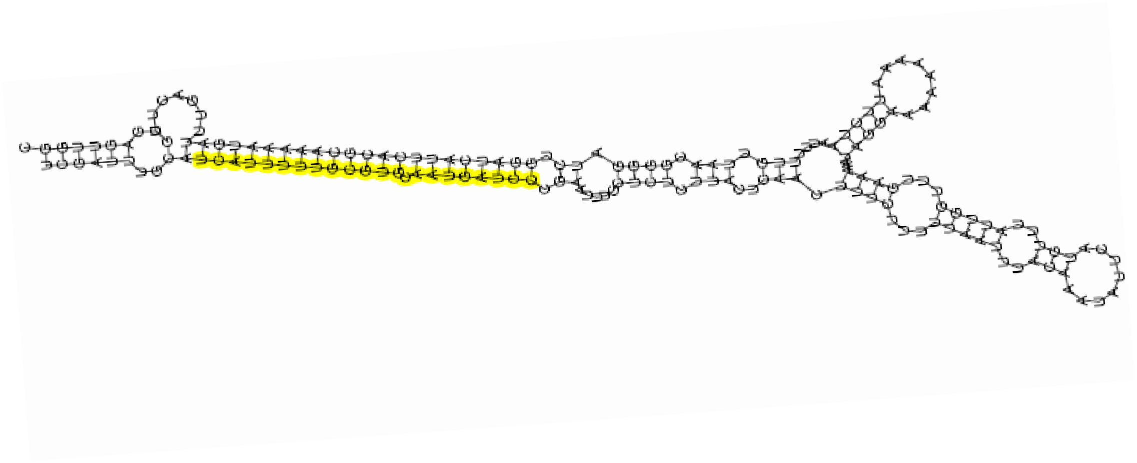

MIR1861a dG = -32.90 kcal/mol TC25920:1352:1475:+ 124(nt)

TGTTATGGCCATGGCCTGCCAAGTTTGCATCACTTCAAGGCTTCTTGGGGCAACGTTTCTAGTTAAAGAGTTTCAGAAAAATGAA
CAAGTTTTGATGTTGAGGCAAAAATGTAGGAGCTATAAT

```
          10        20        30         40          50
U         CAUGG     CAAG       A  -  U           | U GG CA
 GUUAUGGC      CCUGC    UUUGC UC AC UCAAGGCUU--CU G   G  \
 UAAUAUCG      GGAUG    AAACG AG UG AGUUUUGAA  GA C   U   A
-        A----  UAA-     G  U  U          \ ^ U UU GC
   120         110       100      90     60
```

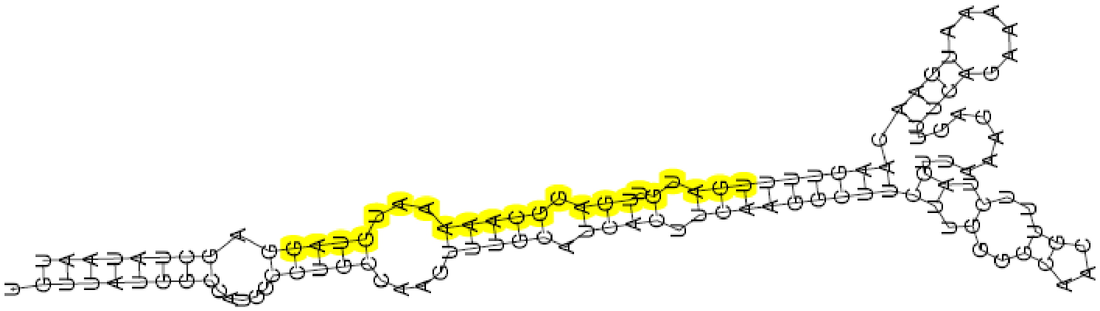

Supplement: Additional file 6 — Fold-back structures for known miRNA from sweet orange (Citrus sinensis). Precursor sequences for known miRNAs from sweet orange were shown in black letters with miRNA and miRNA* (The sequence complementary to miRNA in the fold-back structure) sequences highlighted in yellow and pink, respectively. Precursor secondary structures and dG value were produced using the mfold software http://mfold.bioinfo.rpi.edu/. [file 1471-2164-11-246-S6.PDF]
