# Supplementary material for: Discovery and comparative profiling of microRNAs in a sweet orange red-flesh mutant and its wild type
Source: BMC Genomics. 2010 Apr 17;11:246. doi: 10.1186/1471-2164-11-246 (PMC2864249; doi:10.1186/1471-2164-11-246)

Additional data file 8. Fold-back structures for novel miRNAs from sweet orange (*Citrus sinensis*).

Precursor sequences for novel miRNA from sweet orange were shown in black letters with miRNA and miRNA* (The sequence complementary to miRNA in the fold-back structure) sequences highlighted in yellow and pink, respectively. Precursor secondary structures and dG value were produced using the mfold software (http://mfold.bioinfo.rpi.edu/).

csi-novel-01 dG = -31.80 kcal/mol

AAAACCCAATTTTTTCGGCAACATGATTTCTTAGTAGAAACCAAAATTTACTTTTTGTGCCAACAGGATTTATAATAAGAATTTATGC
AAACGGATTTCTTTTAGAAGTCAAATTTTGACAAGATTGATCATCATCGTTGGTGGGAAACACCAAGTTT

```
          10          20        30          40
.-AAAACC         U   .-CAA     U         |    AAC
        CAAUUUU UCGG      CAUGA UUCUUA--GUAGA    C
        GUUAGAA AGUU      GUAUU AAGAAU  CAUUU    A
\ ------         C   \ ---     U      \ ^     AAA
                120            80      50
```

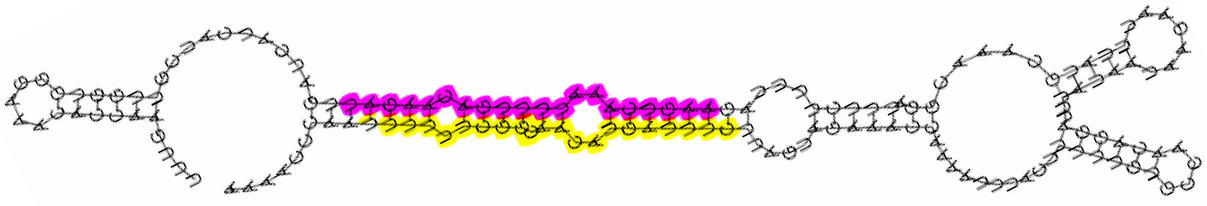

csi-novel-02 dG = -53.10 kcal/mol

AATAGAGAGAGTGAAGTCACTGGAGGCAGCGGTTCATCGATCACTTTGTGCAAATTTTGTTGTGAAAAATAACACAAAATACATGA
ATCGATCGATAAACCTCTGCATCCAGCGCTCACTCCAACTCTATTC

```
           10        20        30        40        50        60
-       A--|     A  CA     G    C    C       AC  UG  CAA       U  GAA
 AAUAGAG   GAGUGA GU  CUGGA GCAG GGUU AUCGAUC  UU  UG   AUUUUGU GU   A
 UUAUCUC   CUCACU CG  GACCU CGUC CCAA UAGCUAG  AA  AC   UAAAACA CA   A
C       AAC^     -  C-     A    U    A       CU  GU  A--       -  AUA
130       120         110       100        90          80         70
```

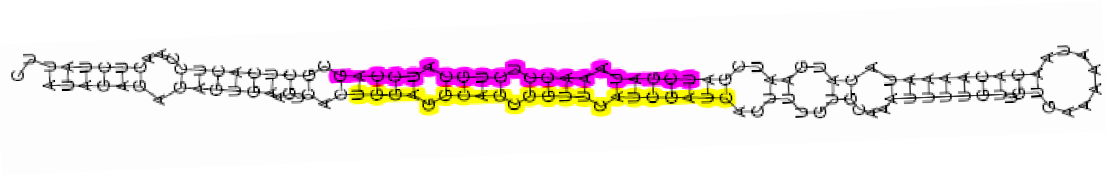

csi-novel-03 dG = -62.40 kcal/mol

CATAAGAGGAGTGCTTGGCATAGATAAAGATGAGAGAAAAATATGAGAGAAGGACGAGAGAAATTTGTTCCCTCTCCACCCACCC
ACTCTCTCTTCTCTTTTCTTCTTTTTCTCTTATCGTTATCTGTGCTTAGCATTTTTCTTCGAT

```
         10        20        30        40        50         60        70
CAU-|           U           A             UAU        -  C      AAUUUGUUCCCU
    AAGAGGAGUGCU GGCAUAGAUAA GAUGAGAGAAAAA   GAGAGAAG GA GAGAGA            C
    UUCUUUUUACGA UCGUGUCUAUU CUAUUCUCUUUUU   UUCUUUUC CU CUCUCU            U
UAGC^           U           G             C--        U  U      CACCCACCCACC
      140       130       120       110         100        90        80
```

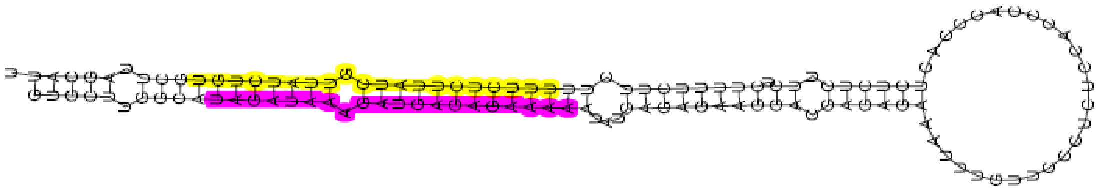

csi-novel-04   dG = -50.00 kcal/mol

CCTGTCATGTTTTTCCACAGCTTTCTTGAACTTCTTTCTTTCTTCTTCTTCTTCTTCTTCTTCTTCTTCTTCTTCTTCTTCTTCTT
CTTCTTCTTCCTCTAATAATAGGCTACGATTTGATGTGGCTAGCTGCAGCTATTAGAAGTTCAAGAAATCTGTGGGAAGAAATGGC
AAA

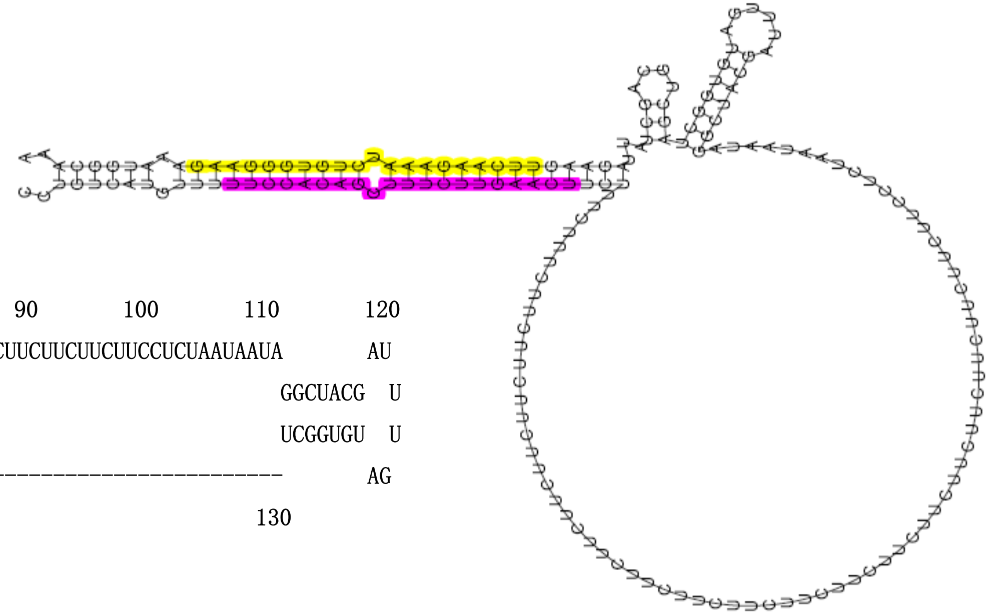

```
        10        20        30          40        50        60        70        80        90       100      110       120
CC|     G           C              .-UUCUUUCUUCUUCUUCUUCUUCUUCUUCUUCUUCUUCUUCUUCUUCUUCUUCUUCUUCUUCUUCUUCCUCUAAUAAUA       AU
  UGUCAU UUUUUCCACAG UUUCUUGAACUUCU                                                                             GGCUACG  U
  ACGGUA AGAAGGGUGUC AAAGAACUUGAAGA                                                                             UCGGUGU  U
AA^     A           U              \ ---------------------------------------------------------------------------       AG
      170       160       150                                                                                 130
```

csi-novel-05-5p R-m0003-5 dG = -60.30 kcal/mol

csi-novel-05-3p R-m0003-3 dG = -60.30 kcal/mol

TATTCTTTCATGTGCTGTAGATAGGCCCTTCAACCTCGGAAACCTCACCATGGACTGGGAGCGAAGGAAGTGGGATCTGATGGTTG
GCATTGTAGGTGGCAAGCTGCAAAAGAAAGATGACGTTTTCAAAGTTGAAGGGCCTTTCTAGAGCACTGGAATCCAT

```
          10        20        30        40          50        60
UAUUC     U      G    U            CUC      C     |   GGA  GGG
     UUUCA GUGCU UAGA AGGCCCUUCAAC   GGAAAC UCA--CCAU   CU   A
     AAGGU CACGA AUCU UCCGGGAAGUUG   CUUUUG AGU  GGUG   GA   G
UACCU     -      G    U            AAA      C   \ ^   AAG  AGC
 160        150       140       130       120      70
```

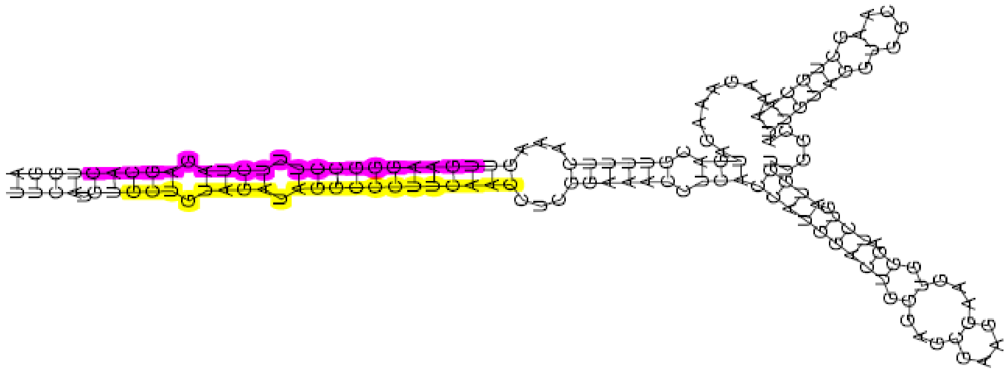

csi-novel-06 R-m0004-5 dG = -36.40 kcal/mol

TAAAATTTTTGGAATTGGGTGCTAGGGAAGGTTTTGTTTGACTTGTCTGTCATAACTAAATTAATTTTCCCTAGTCCCCCTATTCCTAT
GATTTTC

```
         10          20        30              40
U       UUU      U-   UG            UU----   -|   UU
 AAAAUU   GGAAU  GGG  CUAGGGAAGGUU        GUU UGAC  G
 UUUUAG   CCUUA  CCC  GAUCCCUUUUAA        CAA ACUG  U
C       UAU      UC   CU            UUAAAU   U^   UC
      90         80        70        60        50
```

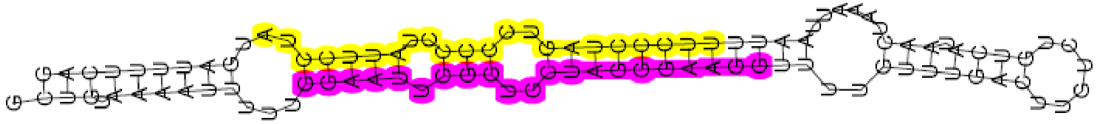

csi-novel-07 **dG = -64.50 kcal/mol**

GGGAGCTTTTTGTTTTGAGGGGAATGTTGTCTGGCTCGAGGACACTGCTTGTTGATCCATTAATTTTACGTATTCTCTCATAGATCTA
GCATCTGAATGGTGGATAACAATATACGGATTCAGCTTATTTGGCGTCGTCGGACCAGGCTTCATTCCCCCCAATATATTGCTTCCAT
GG

```
               10        20        30        40        50             60        70
----      UUUU   U   A         UU     CU   G   A  GCUU--    -    .-AUUAAUUUUACG   UC
    GGGAGC    UGU UUG GGGGAAUG  GUCUGG  CGA GAC CU      GUUG AUCC              UAU  U
    CCUUCG    AUA AAC CCCCUUAC  CGGACC  GCU CUG GG      CGAC UAGG              AUA  C
GGUA      UU--   U   C         UU     AG   G   C  UUUAUU    U    \ ------------   CU
      170         160        150       140       130       120                  80
```

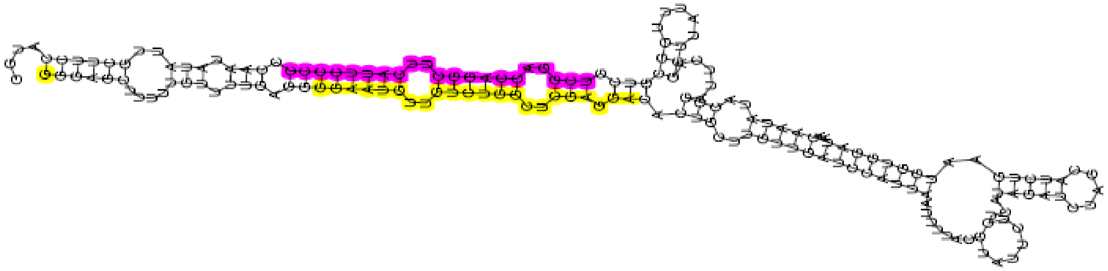

csi-novel-08 dG = -61.80 kcal/mol

TGAAGCTAGGAAGTTTTGGGAGTGGGAGCGTGGGGTAAGAAGAGAAGAAAGCGTATAATTTTTCTTTTCTTTTTCAATTGAGAAAG
AAAATTACAAATTCAATTTCTTCCCTATGCCTCCCATTCCTATGATTTCCTGCAGTTCCT

```
           10         20          30       40            50
UGAA---  U        U           -        U      .-A|       CGU
       GC AGGAAGUU UGGGAGUGGGA GCGUGGGG AAGAAG   GAAGAAAG   A
       CG UCCUUUAG AUCCUUACCCU CGUAUCCC UUCUUU   UUUCUUUU   U
UCCUUGA  -        U           C        -      \ -^       UAA
    140        130       120       110                 60
```

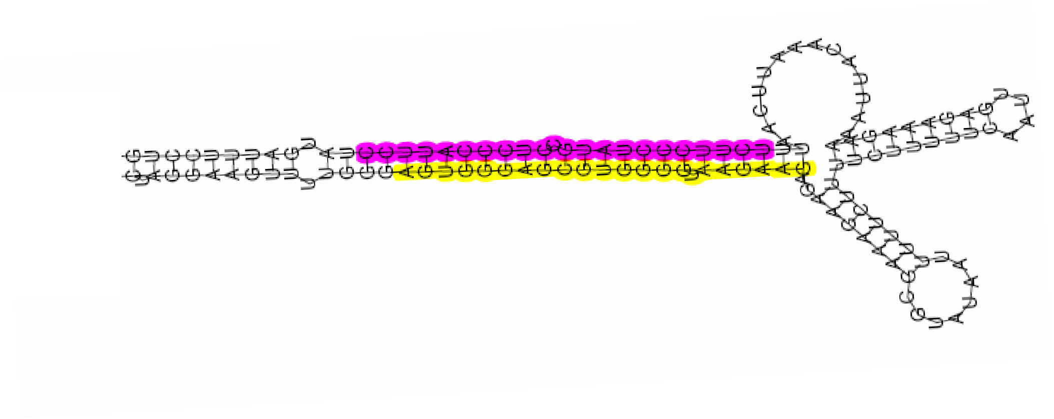

csi-novel-09 dG = -24.40 kcal/mol

TGGATAATCTGATGACTGCAGAGCCCAGACAGAGGCGTCATTCCGTGCATCATAGCCATCACCCCGTCGATTGAGTTCTGCAAGCC
GTCGAGTTTCTGCCT

```
          10         20        30        40        50
U  AU--  UC     A -|       C   ACAGA    UCAUUCCGU  AU
 GG    AA  UGAUG C UGCAGAGC CAG     GGCG         GC  C
 CC    UU  GCUGC G ACGUCUUG GUU     CUGC         CG  A
U  GUCU  GA     C A^       A   AG---    CCCACUAC-  AU
 .        90        80        70           60
```

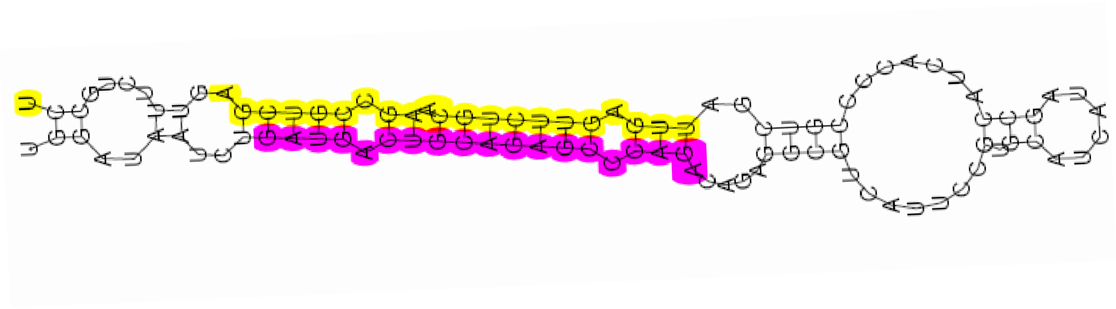

csi-novel-11 dG = -20.80 kcal/mol

TTTACTTGGCTGGACAGAGAAATCACGGTCAAGTCTCTTCTTGTTCAGGAAACGATTGATGATTTCTTCTTCATCTGGTGGATGGAA

```
         10          20         30        40
-    C  G  UG  C--  -        -|    AA  C    CUU
 UUUA UU GC  GA   AG AGAAAUCA CGGUC  GU UCUU   \
 AGGU AG UG  CU   UC UCUUUAGU GUUAG  CA AGGA   G
A    -  G  GU  ACU  U        A^    --  A    CUU
       80         70        60           50
```

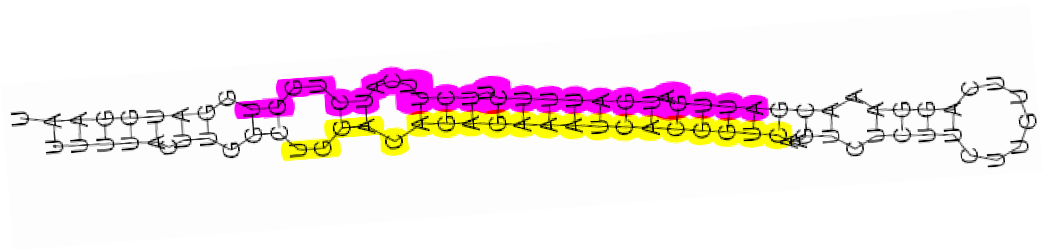

csi-novel-12　　　**dG = -62.20 kcal/mol**

AACAGTCGTTGCTCGCTGTAGCAGCGTCCTCAAGATTCACATCCAGTCTAAAGGCAAAAGCAGCAATTTTTCTTCATTTTTGCTTGC
CTTGGTTTTTGTCAGGGAGAATCTTGATGATGCTGCAACGGCGATTAATGACTAGCTA

```
              10         20         30         40         50         60          70
     AAC--|        C        A         C          ACA    AGUC       AAAA    -     UUUUUC
          AGUCGUUG UCGCUGU GCAGCGUC UCAAGAUUC   UCC    UAAAGGC    GCA GCAA        \
          UCAGUAAU AGCGGCA CGUCGUAG AGUUCUAAG   AGG    GUUUUUG    CGU CGUU        U
     AUCGA^        U        A         U          ---    GACU       GUUC    U     UUUACU
        140        130        120        110          100        90         80
```

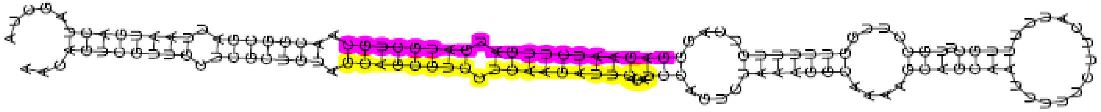

Supplement: Additional file 8 — Fold-back structures for novel miRNAs from sweet orange (Citrus sinensis). Precursor sequences for novel miRNA from sweet orange were shown in black letters with miRNA and miRNA* (The sequence complementary to miRNA in the fold-back structure) sequences highlighted in yellow and pink, respectively. Precursor secondary structures and dG value were produced using the mfold software http://mfold.bioinfo.rpi.edu/. [file 1471-2164-11-246-S8.PDF]
