# Supplementary material for: Discovery and comparative profiling of microRNAs in a sweet orange red-flesh mutant and its wild type
Source: BMC Genomics. 2010 Apr 17;11:246. doi: 10.1186/1471-2164-11-246 (PMC2864249; doi:10.1186/1471-2164-11-246)
Supplement: Additional file 12 — The primer sequence information. This file lists the primers used for miRNA expression detection by stem-loop qRT-PCR. [file 1471-2164-11-246-S12.PDF]

Additional data file 12. Stem loop qRT-PCR primers used in this study

csi-miR156a TGACAGAAGAGAGTGAGCAC
RT-primer：GTCGTATCCAGTGCAGGGTCCGAGGTATTCGCACTGGATACGACGTGCTC
Forward primer： CGGCGTGACAGAAGAGAGT

csi-miR159a TTTGGATTGAAGGGAGCTCTA
RT-primer：GTCGTATCCAGTGCAGGGTCCGAGGTATTCGCACTGGATACGACTAGAGC
Forward primer： CGGAGTTTGGATTGAAGGGA

csi-miR162a TCGATAAACCTCTGCATCCAG
RT-primer：GTCGTATCCAGTGCAGGGTCCGAGGTATTCGCACTGGATACGACCTGGAT
Forward primer： CGAAGTCGATAAACCTCTGC

csi-miR164a TGGAGAAGCAGGGCACGTGCA
RT-primer：GTCGTATCCAGTGCAGGGTCCGAGGTATTCGCACTGGATACGACTGCACG
Forward primer： CAAATTGGAGAAGCAGGGCA

csi-miR166a TCGGACCAGGCTTCATTCCCC
RT-primer：GTCGTATCCAGTGCAGGGTCCGAGGTATTCGCACTGGATACGACGGGGAA
Forward primer： ATTAGTCGGACCAGGCTTCA

csi-miR166j TCTCGGACCAGGCTTCATTCC
RT-primer：GTCGTATCCAGTGCAGGGTCCGAGGTATTCGCACTGGATACGACGGAATG
Forward primer： CTTATTCTCGGACCAGGCTT

csi-miR167d TGAAGCTGCCAGCATGATCTGA
RT-primer：GTCGTATCCAGTGCAGGGTCCGAGGTATTCGCACTGGATACGACTCAGAT
Forward primer： ATTATTGAAGCTGCCAGCATG

csi-miR168a TCGCTTGGTGCAGGTCGGGAA
RT-primer：GTCGTATCCAGTGCAGGGTCCGAGGTATTCGCACTGGATACGACTTCCCG
Forward primer： ATTAGTCGCTTGGTGCAGGT

csi-miR171b CGAGCCGAATCAATATCACTC
RT-primer：GTCGTATCCAGTGCAGGGTCCGAGGTATTCGCACTGGATACGACGAGTGA
Forward primer： CGAAGCGAGCCGAATCAATA

csi-miR172a AGAATCTTGATGATGCTGCAT
RT-primer：GTCGTATCCAGTGCAGGGTCCGAGGTATTCGCACTGGATACGACATGCAG
Forward primer： CGGCGAGAATCTTGATGATG

csi-miR319e TTTGGACTGAAGGGAGCTCCT
RT-primer： GTCGTATCCAGTGCAGGGTCCGAGGTATTCGCACTGGATACGACAGGAGC

Forward primer: GTCAGTTTGGACTGAAGGGA

csi-miR390a AAGCTCAGGAGGGATAGCGCC
RT-primer：GTCGTATCCAGTGCAGGGTCCGAGGTATTCGCACTGGATACGACGGCGCT
Forward primer： CAATGAAGCTCAGGAGGGAT

csi-miR395a CTGAAGTGTTTGGGGGAACTC
RT-primer：GTCGTATCCAGTGCAGGGTCCGAGGTATTCGCACTGGATACGACGAGTTC
Forward primer： CAATGCTGAAGTGTTTGGGG

csi-miR403 TTAGATTCACGCACAAACTCG
RT-primer：GTCGTATCCAGTGCAGGGTCCGAGGTATTCGCACTGGATACGACCGAGTT
Forward primer： CGGAGTTAGATTCACGCACA

csi-miR472a TTTTCCCACACCTCCCATCCC
RT-primer：GTCGTATCCAGTGCAGGGTCCGAGGTATTCGCACTGGATACGACGGGATG
Forward primer： CGCAGTTTTCCCACACCTCC

csi-miR473a ACTCTCCCTCAAGGGCTTCGC
RT-primer：GTCGTATCCAGTGCAGGGTCCGAGGTATTCGCACTGGATACGACGCGAAG
Forward primer： CTTATACTCTCCCTCAAGGG

csi-miR477a ACCTCCCTCGAAGGCTTCCAA
RT-primer：GTCGTATCCAGTGCAGGGTCCGAGGTATTCGCACTGGATACGACTTGGAA
Forward primer： ATTATACCTCCCTCGAAGGC

csi-miR479 TGTGATATTGGTTCGGCTCATC
RT-primer：GTCGTATCCAGTGCAGGGTCCGAGGTATTCGCACTGGATACGACGATGAG
Forward primer： CAATGTGTGATATTGGTTCGG

csi-miR482a TCTTCCCTATGCCTCCCATTCC
RT-primer：GTCGTATCCAGTGCAGGGTCCGAGGTATTCGCACTGGATACGACGGAATG
Forward primer： ATTATTCTTCCCTATGCCTCC

csi-miR482c TCTTGCCCACCCCTCCCATTCC
RT-primer： GTCGTATCCAGTGCAGGGTCCGAGGTATTCGCACTGGATACGACGGAATG
Forward primer： ATTTCTTGCCCACCCCTCC

csi-novel-01 (CK07-5) TTTTTCGGCAACATGATTTCT
RT-primer：GTCGTATCCAGTGCAGGGTCCGAGGTATTCGCACTGGATACGACAGAAAT
Forward primer： CGGCATTTTTCGGCAACATG

csi-novel-02 (CK14-3) TTCAAGAAATCTGTGGGAAG

RT-primer：GTCGTATCCAGTGCAGGGTCCGAGGTATTCGCACTGGATACGACCTTCCC
Forward primer： CGGCGTTCAAGAAATCTGT

csi-novel-03 (R03-5) GCTGTAGATAGGCCCTTCAAC
RT-primer：GTCGTATCCAGTGCAGGGTCCGAGGTATTCGCACTGGATACGACGTTGAA
Forward primer： CTTATGCTGTAGATAGGCCC

csi-novel-03-3p (R03-3) TGAAGGGCCTTTCTAGAGCAC
RT-primer：GTCGTATCCAGTGCAGGGTCCGAGGTATTCGCACTGGATACGACGTGCTC
Forward primer： CGGATTGAAGGGCCTTTCTA

csi-novel-04 (R04-5) GGAATTGGGTGCTAGGGAAGG
RT-primer：GTCGTATCCAGTGCAGGGTCCGAGGTATTCGCACTGGATACGACCCTTCC
Forward primer： ATACGGGAATTGGGTGCTAG

csi-novel-04-3p (R04-3) TTCCCTAGTCCCCCTATTCCTA
RT-primer：GTCGTATCCAGTGCAGGGTCCGAGGTATTCGCACTGGATACGACTAGGAA
Forward primer： ATTATTTCCCTAGTCCCCCTA

csi-novel-05 (R06-5) GGAATGTTGTCTGGCTCGAGG
RT-primer：GTCGTATCCAGTGCAGGGTCCGAGGTATTCGCACTGGATACGACCCTCGA
Forward primer： CGATTGGAATGTTGTCTGGC

csi-novel-6 (R08-5) AGTGGGAGCGTGGGGTAAGAAG
RT-primer：GTCGTATCCAGTGCAGGGTCCGAGGTATTCGCACTGGATACGACCTTCTT
Forward primer： ATATAGTGGGAGCGTGGGGT

csi-novel-7 (R09-5) TGGAGGCAGCGGTTCATCGATC
RT-primer：GTCGTATCCAGTGCAGGGTCCGAGGTATTCGCACTGGATACGACGATCGA
Forward primer： ATTATGGAGGCAGCGGTTCA

csi-novel-8 (R10-5) TAGATAAAGATGAGAGAAAAA
RT-primer：GTCGTATCCAGTGCAGGGTCCGAGGTATTCGCACTGGATACGACTTTTTC
Forward primer： CGGCGTAGATAAAGATGAGA

csi-novel-8-3p (R10-3) TTTCTCTTATCGTTATCTGT
RT-primer：GTCGTATCCAGTGCAGGGTCCGAGGTATTCGCACTGGATACGACACAGAT
Forward primer： CGGCGTTTCTCTTATCGTT

csi-novel-9 (R12-3) TTGAGTTCTGCAAGCCGTCGA
RT-primer：GTCGTATCCAGTGCAGGGTCCGAGGTATTCGCACTGGATACGACTCGACG
Forward primer： ATCCGTTGAGTTCTGCAAGC

csi-novel-10 (R14-3) AGGTCATCTTGCAGCTTCAAT

RT-primer：GTCGTATCCAGTGCAGGGTCCGAGGTATTCGCACTGGATACGACATTGAA

Forward primer： CGAATAGGTCATCTTGCAGC

csi-novel-11 (R19-5) TGGACAGAGAAATCACGGTCA

RT-primer：GTCGTATCCAGTGCAGGGTCCGAGGTATTCGCACTGGATACGACTGACCG

Forward primer： ACGCGTGGACAGAGAAATCA

csi-novel-12 (R21-5) GCAGCGTCCTCAAGATTCACA

RT-primer：GTCGTATCCAGTGCAGGGTCCGAGGTATTCGCACTGGATACGACTGTGAA

Forward primer： CTTATGCAGCGTCCTCAAGA

Reverse primer : GTGCAGGGTCCGAGGT
